# *De Novo* Transcriptome Analysis of Medicinally Important *Plantago ovata* Using RNA-Seq

**DOI:** 10.1371/journal.pone.0150273

**Published:** 2016-03-04

**Authors:** Shivanjali Kotwal, Sanjana Kaul, Pooja Sharma, Mehak Gupta, Rama Shankar, Mukesh Jain, Manoj K. Dhar

**Affiliations:** 1 School of Biotechnology, University of Jammu, Jammu, Jammu & Kashmir, 180006, India; 2 National Institute of Plant Genome Research (NIPGR), Aruna Asaf Ali Marg, New Delhi, 110067, India; CSIR-National Botanical Research Institute, INDIA

## Abstract

*Plantago ovata* is an economically and medicinally important plant of the family Plantaginaceae. It is used extensively for the production of seed husk for its application in pharmaceutical, food and cosmetic industries. In the present study, the transcriptome of *P*. *ovata* ovary was sequenced using Illumina Genome Analyzer platform to characterize the mucilage biosynthesis pathway in the plant. *De novo* assembly was carried out using Oases followed by velvet. A total of 46,955 non-redundant transcripts (≥100 bp) using ~29 million high-quality paired end reads were generated. Functional categorization of these transcripts revealed the presence of several genes involved in various biological processes like metabolic pathways, mucilage biosynthesis, biosynthesis of secondary metabolites and antioxidants. In addition, simple sequence-repeat motifs, non-coding RNAs and transcription factors were also identified. Expression profiling of some genes involved in mucilage biosynthetic pathway was performed in different tissues of *P*. *ovata* using Real time PCR analysis. The study has resulted in a valuable resource for further studies on gene expression, genomics and functional genomics in *P*. *ovata*.

## Introduction

*Plantago* is an important genus on which family Plantaginaceae is based [1]. These plants are commonly known as Plantains and are mostly annual or perennial herbs or sub-shrubs. Only two, namely *P*. *ovata* and *P*. *psyllium* have been extensively used for the production of seed husk out of more than 200 species of the genus. Psyllium seed has been used in traditional medicine since long. It is well renowned for its mucilaginous property, which is due to the seed husk [2]. The seed husk is colorless and is commonly known as “Isabgol” in Hindi and “Blonde Psyllium” in English. India holds monopoly in the world trade of Psyllium, which is cultivated on a large scale in North Gujarat. *P*. *ovata* has a narrow genetic base on account of small, mostly heterochromatic chromosomes with low chiasmata frequency and recombination index. It is a diploid (2n = 2x = 8) plant with a genome size of about 621 Mb [3].

Psyllium has been extensively studied in view of its several health benefits and applications in pharmaceutical, food and cosmetic industry. Apart from its recognized laxative property, Psyllium has other potential benefits like lowering of blood cholesterol and hyperglycemia, reducing the risk of colon cancer, ulcerative colitis and treatment of irritable bowel syndrome [2, 3]. It has been used as a deflocculant in paper and textile manufacturing, as an emulsifying agent, as binder or lubricant in meat products, and as a replacement of fat in low-calorie foods. It has also been incorporated into breakfast cereals, ice creams, instant beverages, bakery and other dietary products [2, 4].

Transcriptome analysis enables to understand genome expression at transcript level, hence, providing information on gene structure, regulation of gene expression, its function and genome dynamics [5]. With the advent of next-generation sequencing (NGS) technologies gene discovery via RNA sequencing has become rapid and cost-effective. Since the sequence reads generated from the high throughput-sequencing platforms are shorter in length than classical Sanger sequences, therefore, it is necessary to reconstruct the full length transcripts by transcriptome assembly [6, 7].

**Fig 1:**
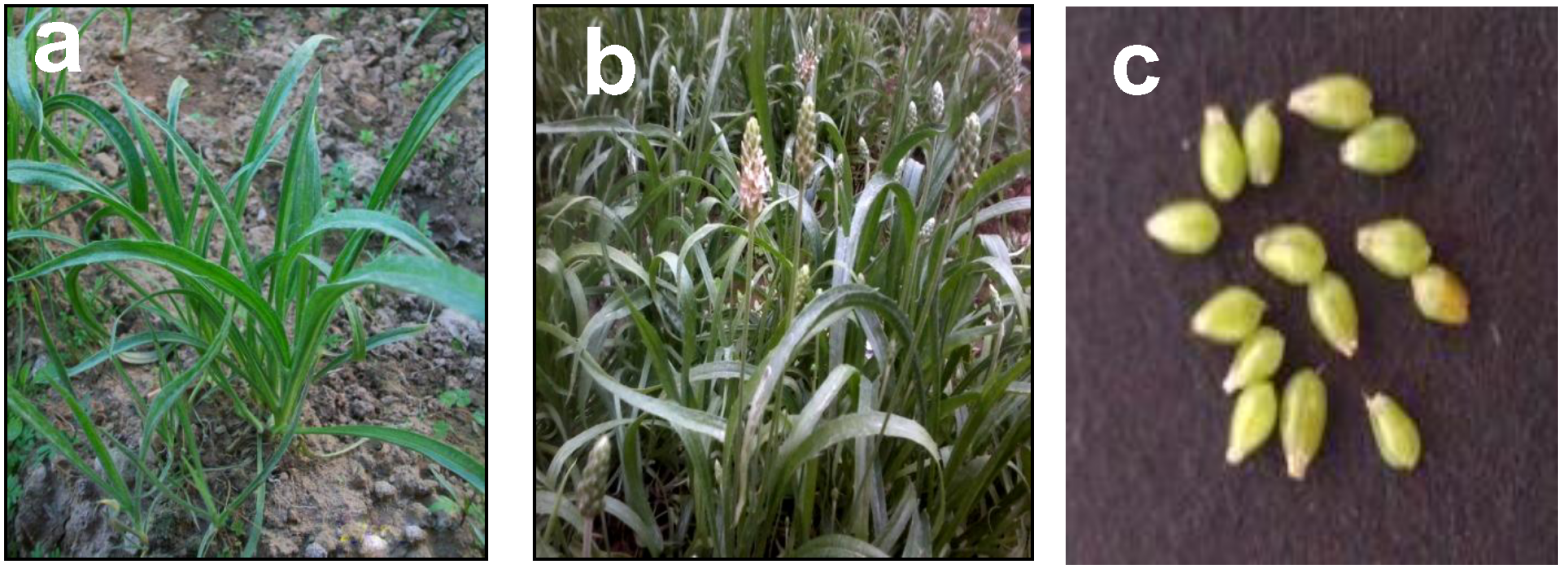
*Plantago ovata* plant at (a) vegetative and (b) reproductive phase, (c) *P*. *ovata* ovaries.

*P*. *ovata* is one of the important medicinal and commercial plants in India (Fig 1a and 1b). Although, there are some reports on genetic characterization of this species, it is very essential to develop genomic and transcriptomic resources for its further genetic improvement. One of the most important properties of seed husk (Isabgol) is that it absorbs the water and releases mucilage. The seed coat consists of mucilage producing cells (MPCs), filled with mucilage [2]. Rapid cell expansion and differentiation starts with pollination. As the ovary matures into a seed, MPCs undergo a complex differentiation process leading to thin walled containers of almost pure mucilage [8]. Despite being medicinally important, there are no reports on the characterization of mucilage biosynthetic pathway in this species. Thus, in order to mine the genes associated with the mucilage pathway, developing ovaries were selected for the transcriptome analysis. We devised a strategy to perform *de novo* assembly of transcriptome using short-read sequence data.

Total unigenes were used for functional categorizations and discovery of various transcription factor families. GO analysis and pathways analysis were also carried out to discover various processes and pathways involved in biosynthesis of medicinally important compounds. In addition, GC content analysis, non-coding RNAs (ncRNAs) and simple sequence repeats (SSRs) were also identified to understand the genome complexity of this plant. The data generated from this study has resulted in valuable genetic resource which can be utilized to improve the medicinal properties by modifying the underlying processes/pathways.

## Materials and Methods

### Plant material and RNA isolation

Seeds of *Plantago ovata* were sown during second week of October in experimental plots in the Botanical Garden, University of Jammu. For the present study, ovaries were collected at different stages of seed development (0, 1, 2, 3, 4, 5, 6, 7, 10, 15, 20, 25 days after pollination) (S1 Table and S1 Fig). Mucilage content was estimated by following the method detailed in Sharma and Koul [9]. Total RNA was isolated using TRIzol^®^ reagent (Life Technologies, Carlsbad, CA) according to manufacturer’s instructions. Nanodrop 2000 (Thermo Fisher Scientific, Wilmington, DE) was used for quantitative and qualitative analysis of the RNA samples.

### Illumina sequencing and quality control

A total of 10 μg of total RNA (pooled in equal quantity from three biological replicates) was used for library preparation and sequencing. Libraries were prepared according to Illumina TrueSeq RNA library method as per “TrueSeq RNA Sample preparation guide” (Illumina Technologies). 72 bp PE sequencing was carried out using Illumina Genome Analyzer II platform. The raw sequence data obtained after sequencing was made to undergo quality control screening by using NGS QC Toolkit [10] to remove the low quality reads and reads containing primer/adapter sequences. The sequence data generated in this study have been deposited at NCBI in the Short Read Archive database under the accession number SRP017437.

### De novo assembly

The high quality reads were used for *de novo* assembly. All the assemblies were performed on a server with 48 cores and 256 GB random access memory. Publically available programs like Velvet (v1.2.07) [11], Oases (v0.2.08) [12], ABySS (v1.2.7) [13] and commercially available CLC Genomics workbench (v4.7.2) were used for *de novo* assembly. Velvet, Oases and ABySS were run at various *k*-mer to optimize the assembly. Oases package operates on the output of the Velvet assembler, utilizing the pairing information in the sequencing reads to identify and group transcript isoforms into appropriate loci.

### Similarity search and functional annotation

Due to non-availability of any reference genome, proteome data sets of twenty five completely sequenced plant genomes, belonging to fifteen different families, were retrieved from Phytozome v9.1 (www.phytozome.net). BLASTX search of *P*. *ovata* transcripts against these proteome sequences with an E-value ≤1e-05 was carried out to identify sequence conservation. Further, to deduce and assign putative function, thetranscripts were subjected to BLASTX search against non-redundant (Nr) database of NCBI, UniRef100, UniRef90, UniRef 50, pfam, Swiss-Prot, TrEMBL and Conserved domain (CD) database with an e-value ≤1e-05.

KOG, KEGG (v70.0) [14], GO slim terms for categories: molecular function, biological process and cellular component associated with the best BLASTX hit with *Arabidopsis thaliana* proteome were assigned to the corresponding transcripts of *P*. o*vata* using Blast2GO program [15].

For the identification of transcription factor families represented in present data set, the transcripts were searched against all the transcription factor protein sequences present in Plant transcription factor database [16] using BLASTX with an E-value of ≤1e-10.

### GC content analysis, SSR identification and identification of noncoding RNA

GC content of *Plantago ovata* unigenes was analyzed by using custom perl scripts. To provide a reference, GC content range of transcripts of *A*. *thaliana*, tomato and *Eucalyptus* (dicots) along with rice (monocot) was also determined. To identify SSRs in *P*. *ovata*, a perl script program MISA (MicroSAtellite; http://pgrc.ipk-gatersleben.de/misa/) was used. For search criteria, minimum unit size for tri- to hexa-nucleotide repeats was set at five and for di-nucleotide repeat; six was set as the minimum unit size. The noncoding RNAs (ncRNAs) were identified in the *P*. *ovata* transcriptome using Repeat Masker (v4.0.5) with default parameters.

### Quantitative real-time PCR (qRT-PCR)

During the present investigation, expression of the genes involved in mucilage biosynthetic pathway was also studied by two step qRT-PCR. To perform qPCR experiments, RNA isolation was carried out followed by First strand cDNA synthesis. Gene specific qRT-PCR primers were designed using Primer Express Software v2.0. The primers used are presented in S2 Table. The qPCR was performed using Power SYBR^®^Green PCR Master Mix (Applied Biosystems) in ABI 7500 Thermal cycler (Applied Biosystems, Foster City, USA). The qPCR cycling was performed at 50°C for 2 min, 10 minutes polymerase activation at 95°C and 40 cycles at 95°C for 15 seconds and 60°C for 1 min and finally a dissociation stage (melt curve) at 95°C for 15 seconds, 60°C for 1 min and 95°C for 15 seconds. Three biological replicates were used. Amplicons were subjected to the melt curve analysis to check the specificity of the amplified products. The relative expression level of each gene was calculated by the 2^−(ΔΔCt)^ [17] and actin gene was used as housekeeping gene to normalize the amount of template cDNA added in each reaction.

## Results and Discussion

The advent of next generation sequencing has created new avenues for generation of voluminous sequence information in terms of genomic and transcriptomic data. The data is becominguseful in inferring the basic biological, molecular and cellular processes for non-model organisms and non-sequenced genomes [18–20]. By comparing the mucilage content in the developing ovaries, it was observed that mucilage production is at its peak in ovaries between 15–20 days after pollination (DAP) (Dhar et al., Unpublished data). In the present case, ovaries at 15 DAP (Fig 1c) stage were selected, as at this stage, differentiation of the mucilage producing cells (MPCs) would have started, which may lead to activation of the genes involved in the mucilage biosynthetic pathway. Also, at this developmental stage, expression of few of these genes could be expected; hence, mining would be easier. Another aspect which prompted the selection of ovaries at 15 DAP stage was that when RNA was isolated from the ovaries at a later developmental stage (when they headed towards maturity), the mucilage content in the tissue had increased so much that it hindered the RNA extraction. Therefore, three independent biological replicates of the developing ovary tissue 15 DAP were harvested for total RNA isolation.

### Sequence quality controls and preprocessing

In the present experiment, a total of 31,280,458 PE sequence reads (15,640,229 from each side), 72 bp in length and encompassing 6.9 GB sequence data were generated. Using NGS QC tool kit, a total of 29,861,418 (95.46%) high quality filtered reads were generated which were used for the optimization of *de novo* assembly and the analysis of *Plantago ovata* transcriptome (S2 Fig, Table 1). Reads with average Phred score of ≥30 were considered as the high quality reads (S3 Fig). According to Garg et al. [21], program parameters optimization and removing of low quality reads is a necessary practice to improve the assembly output significantly.

**Table 1:** QC summary of the paired end sequencing data.

File name	Paired end library
Total number of reads	31,280,458
Total number of bases	2,252,192,976
Number of primer/ adapter contaminated HQ reads	270
Total number of HQ filtered reads	29,861,418
Percentage of HQ filtered reads	95.46%

### Generation of De novo assembly

Optimization of different assembly programs is essential to obtain the desired results. The high quality sequence reads were assembled by using four different assemblers namely Velvet, Oases, AbySS and CLC genomics workbench. Velvet and Oases were used to assemble the reads at different *k*-mer lengths from 27 to 69, whereas, in ABySS, *k*-mer lengths 29–53 were used and CLC genomics workbench (with default parameters) was used for *de novo* assembly. Analysis of various parameters like total number of contigs, minimum transcript length, average transcripts length, N50 length, which depends on the *k*-mer length, was also performed (S3 Table).

**Fig 2:**
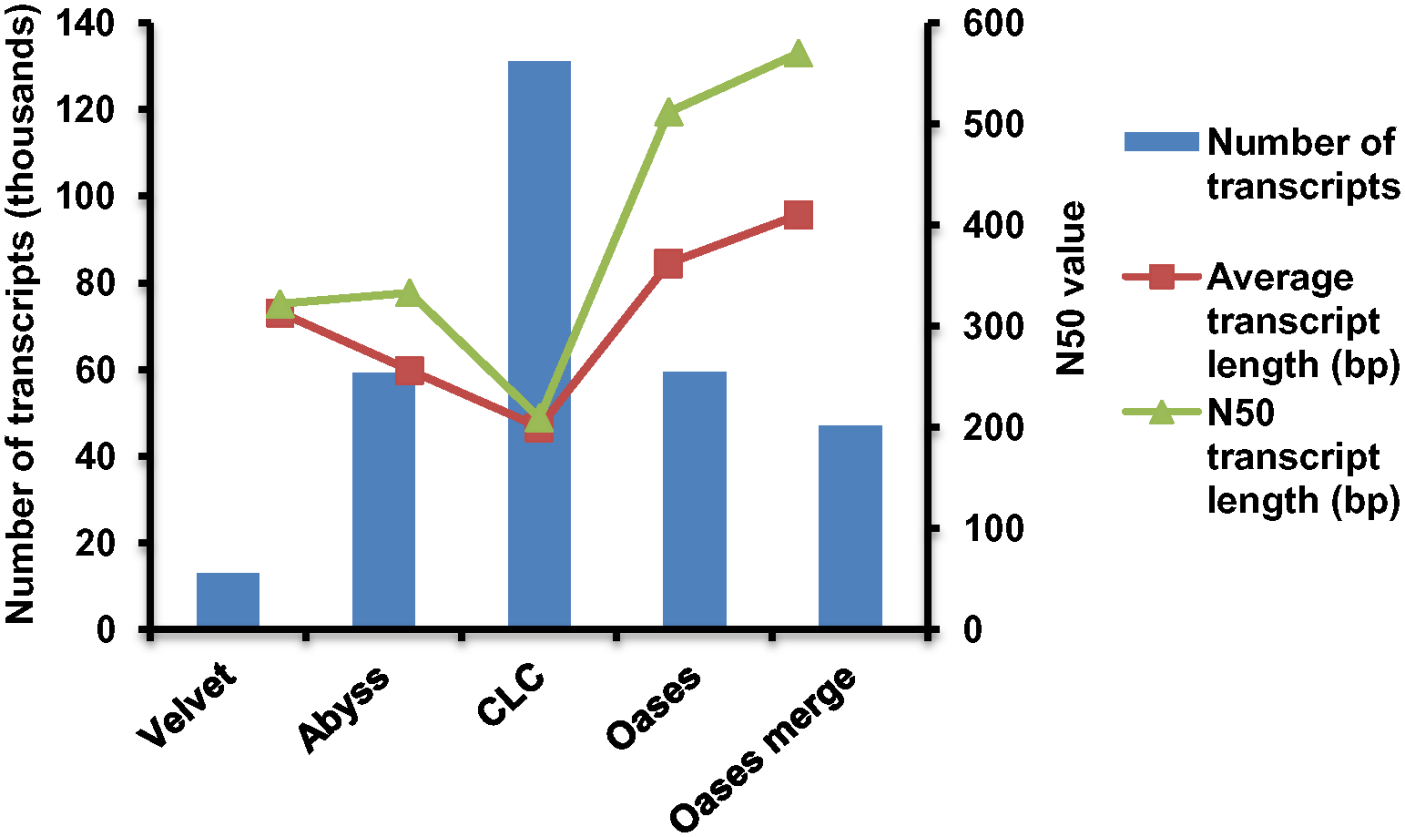
Comparison of *de novo* assembly of the data obtained through Velvet, Abyss, CLC genomics workbench, Oases and merged assembly of Oases programs. The merged assembly produces less number of transcripts but the N50 transcript length and Average transcript length is much higher as compare to others.

Best assembly using Velvet was obtained at *k*-mer length 59 where number of transcripts generated, average transcript length and N50 length were 12,979; 313.35 and 322, respectively (S4 Fig). AbySS gave best assembly with total number of transcripts, average transcript length and N50 value of 59,200; 256.06 and 333 at *k*-mer 49 (S4 Fig). A total of 131,188 transcripts with average transcript length of 199.79 and N50 length of 209 were obtained from assembly with CLC genomics workbench. Assembly of Oases was far better than velvet, ABySS and CLC workbench. The best assembly from Oases was obtained with *k*-mer length 35 where number of transcripts was 59,351; average transcript length and N50 value were 362.07 and 512, respectively (S4 Fig). An increase in N50 length and average transcript length was observed with an increase in *k*-mer length from 27 to 41, beyond which a drop in these values was noticed. Merging of assemblies from *k*-mer 31 to 41 was carried out using an Oases pipeline and output was filtered to remove various isoforms of a particular locus. The longest transcript isoforms were considered to be better as compared to individual assemblies at a particular *k*-mer, with an average transcript length and N50 length of 410 bp and 570 bp, respectively. A total of 46,955 unigenes generated by the merged assembly were used for further analysis (Fig 2) (Table 2). Analysis of the length distribution of the final assembled transcripts was also performed and it was observed that 33% of the total transcripts lie into the range of 100–200 bp length. Transcripts with length >1,500 bp were higher as compared to the transcripts falling in the range of 1,100 to 1,400 bp (S5 Fig).

**Table 2:** Assembled transcripts obtained after merging of Oases assembly with k-mer lengths 31–41.

Parameters	Longest isoforms
Number of transcripts	46,955
Total bases	19,251,438
Minimum transcript length	100
Maximum transcript length	3,544
Average transcript length	410
N50 length	570

### GC content analysis for *P*. *ovata* transcriptome

GC content analysis provides an insight into evolution, thermostability, gene structure (intron size and number) and gene regulation. It is an important criterion for establishing phylogenetic and evolutionary relationships among various species [22, 23]. Focusing on GC poor and homogenous *A*. *thaliana* and much more GC rich (rice) genome has often been generalized as dicot/ monocot dichotomy.

**Fig 3:**
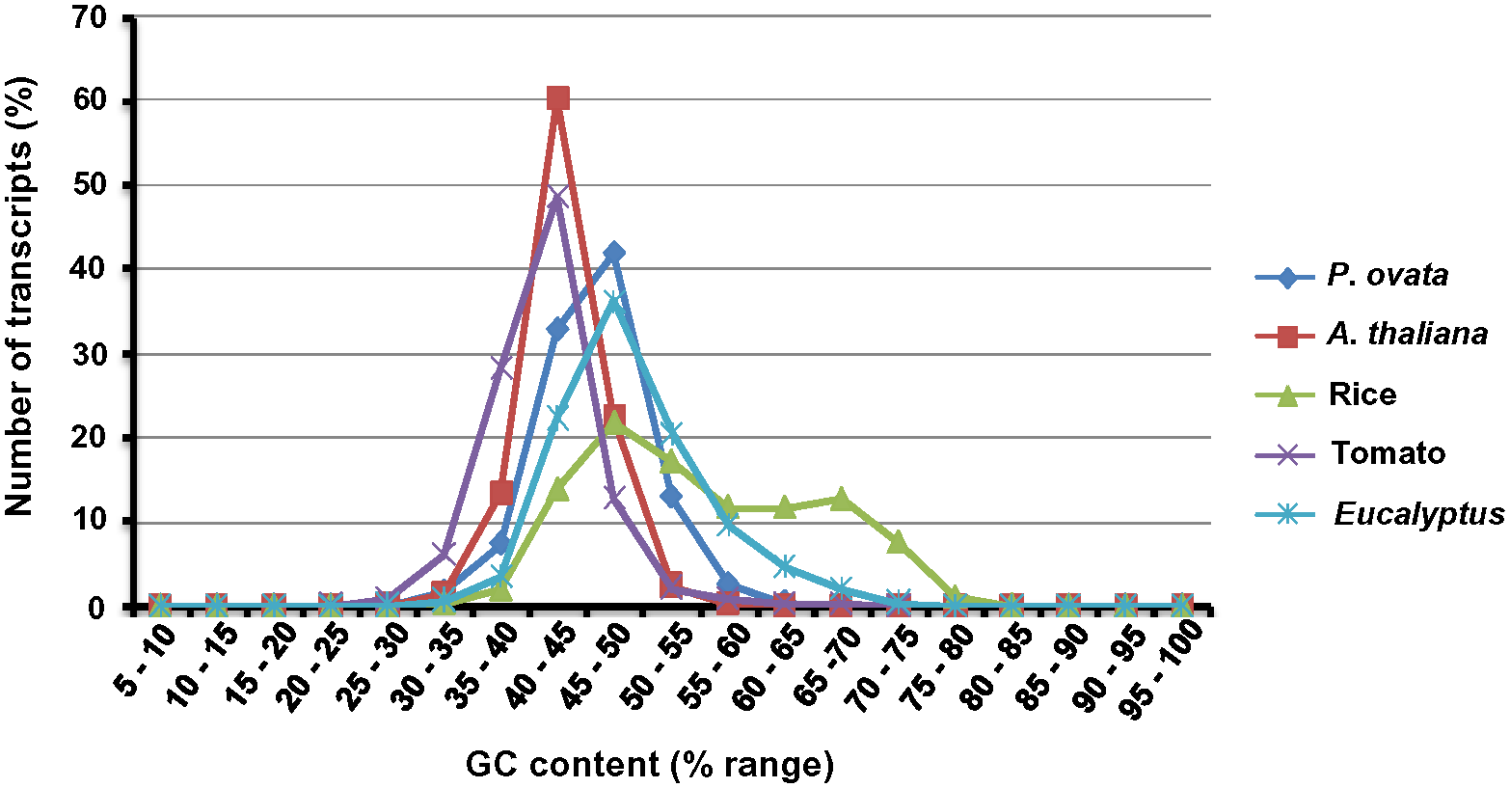
Percentage GC content of *P*. *ovata*, *A*. *thaliana*, rice, tomato and *Eucalyptus* transcripts. The percentage GC content of *P*. *ovata* and *Eucalyptus* falls into the range of monocots.

Analysis of the ratio of guanine and cytosine (GC content) of unigenes in present case, along with transcript sets of four different plants, revealed that the *P*. *ovata* (most of the transcripts, 41.94%) and *Eucalyptus* being eudicot, fall under GC content range of 45–50% along with a monocot, rice. On the other hand, *A*. *thaliana* and tomato have the GC content in the range of 40–45% (Fig 3). Giardi et al. [24] and Mudalkar et al. [18] reported GC content of dicots *Eucalyptus grandis* and *Camelina sativa* to be higher as compared to that of monocots. The current study also reports *P*. *ovata* to have a higher GC content.

Dhar et al. [3] determined 59.7% AT content of *P*. *ovata* by flow cytometry. The GC content by this method turned out to be 40.3%. This finding is not in consonance with the GC content obtained in the present transcriptome analysis. This mismatch can be explained by the fact that the study by Dhar et al. [3] included genomic DNA wherein, both exonic and intronic regions were taken into consideration for determination of GC content, whereas, the present investigation was based on transcriptomic data covering only exonic portion. Differences in the proportion of coding and non-coding DNA perhaps contribute to the variability in GC content. Generally, genes and gene rich regions have been observed to be much more GC rich than non-coding ones [25]. This further helps in explaining the difference in the GC content of the genome and the transcriptome of *P*. *ovata*.

### Similarity search and functional annotation

In order to assess and annotate the assembled unigenes of *Plantago ovata*, the proteome of 25 sequenced plant genomes were retrieved from Phytozome database. The *P*. *ovata* transcripts were searched against proteome sequences of each plant using BLASTX search with an e value ≤1e-5. Overall 28,160 (60%) of the transcripts showed significant similarity with at least one protein sequence from 25 plant species. The largest number (58.7%) of *P*. *ovata* transcripts showed significant similarity with *Mimulus guttatus* (Scrophulariaceae) proteome followed by *Solanum lycopersicum* (Solanaceae, 56.7%). Our findings are in agreement with the studies of Olmstead et al. [26], Albach et al. [27] and Passarin et al. [28], which documented the phylogenetic closeness of the family Plantaginaceae with Scrophulariaceae and Solanaceae. *P*. *ovata* unigenes also showed sequence similarity with *Theobroma cacao* (56.1%); *Poplar trichocarpa* (55.2%); *Prunus persica* (55.3%); *Ricinus communis* (54.9%); *Glycine max* (54.7%); *Phaseolus vulgaris* (54.5%); *Vitis vinifera* (54.4%) and least similarity with *Zea mays* (48.7%) among the monocots (Table 3).

**Table 3:** Number of unigenes showing sequence homology with proteomes of twenty fiveplants with an e-value ≤1e -5 and ≥80% coverage.

Plant reference	Family	1E-5 cut off	> = 80% coverage
*Arabidopsis thaliana*	Brassicaceae	24,866 (52.95%)	2,184 (4.65%)
*Arabidopsis lyrata*	Brassicaceae	24,561 (52.30%)	2,109 (4.49%)
*Brassica rapa* (Chinese cabbage)	Brassicaceae	24,659 (52.51%)	2,194 (4.67%)
*Solanum lycopersicum* (Tomato)	Solanaceae	26,626 (56.70%)	2,351 (5.0%)
*Solanum tuberosum* (Potato)	Solanaceae	24,080 (51.28%)	2,656 (5.65%)
*Glycine max* (Soyabean)	Fabaceae	25,718 (54.77%)	2,293 (4.88%)
*Medicago truncatula*	Fabaceae	25,366 (54.02%)	2,189 (4.66%)
*Phaseolus vulgaris* (common bean)	Fabaceae	25,633 (54.59%)	2,251 (4.79%)
*Prunus persica* (Peach)	Rosaceae	26,010 (55.39%)	2,222 (4.73%)
*Malus domestica* (Apple)	Rosaceae	25,060 (53.37%)	1,659 (3.53%)
*Fragaria vesca* (Strawberry)	Rosaceae	25,294 (53.86%)	1,530 (3.25%)
*Ricinus communis* (Castor bean)	Euphorbiaceae	25,789 (54.92%)	2,237 (4.76%)
*Carica papaya* (Papaya)	Caricaceae	24,609 (52.40%)	2,287 (4.87%)
*Vitis vinifera* (Grape)	Vitaceae	25,566 (54.44%)	2,113 (4.50%)
*Cucumis sativus* (Cucumber)	Cucurbitaceae	25,141 (53.54%)	2,277 (4.84%)
*Theobroma cacao* (Cocoa tree)	Malvaceae	26,354 (56.12%)	2,165 (4.61%)
*Linum usitatisimum* (Flax)	Linaceae	24,928 (53.08%)	2,007 (4.27%)
*Citrus sinensis* (Sweet orange)	Rutaceae	25,388 (54.06%)	2,495 (5.31%)
*Populus trichocarpa* (Poplar)	Saliaceae	25,941 (55.24%)	2,370 (5.04%)
*Eucalyptus grandis*	Myrtaceae	25,380 (54.05%)	2,084 (4.43%)
*Mimulus guttatus*	Scrophulariaceae	27,604 (58.76%)	2,482 (5.28%)
*Brachipodium distyachiyon*	Poaceae	23,454 (49.94%)	1,957 (4.16%)
*Oryza sativa* (Rice)	Poaceae	23,088 (49.17%)	1,914 (4.07%)
*Zea mays* (Maize)	Poaceae	22,875 (48.71%)	2,115 (4.50%)
*Sorghum bicolor*	Poaceae	23,396 (49.82%)	1,848 (3.93%)

BLASTX similarity search against non-redundant (Nr) database and several other databases namely UniRef100, UniRef90, UniRef50, Pfam, Swiss-Prot, TrEMBL, Conserved Domain Database (CDD) also provided an insight into the complex metabolic pathways and regulatory networks that were elucidated by transfer of information and knowledge from the already characterized and annotated genomes to *P*. *ovata* unigenes. A total of 61.6% (28,929 out of 46,955) *P*. *ovata* unigenes could be functionally annotated. This number is comparatively higher than that of other plants like *Sophora japonica* [20], Seabuckthorn (*Hippophae rhamnoides*) [29] and Safflower [30]. The remaining 18,026 unigenes did not show significant similarity with any of the data analyzed. This may be due to novel genes, which perform particular plant specific function or highly divergent genes, or these unigenes could represent untranslated regions.

### Functional classification by GO

Gene ontology (GO), an international standardized gene functional classification system, is a useful tool to annotate large number of genes and their products and analyze their functions [31]. GO terms were assigned to *P*. *ovata* transcripts which showed significant similarity with *A*. *thaliana* proteins annotated with GO terms. GO classification based on sequence homology revealed that 7,790 transcripts out of 22,407 (transcripts showing similarity with *A*. *thaliana*) could be grouped and distributed under three main categories, namely molecular function (2,362; 30.3%), biological process (1,899; 24.3%) and cellular components (3,529; 45.3%).

Within the molecular function category, genes encoding protein binding (38.3%) and proteins related to transferase activity (9.1%) were the most enriched, followed by kinase activity (6.9%), catalytic activity (6.2%), oxidoreductase (4.9%), hydrolase activity (4.8%), transporter activity (4.2%), peptidase activity (3.6%), ligase activity (2.0%) and phosphatase activity (1.7%), which were also significantly represented. The large number of these annotated enzymes with the listed groups suggests the presence of genes associated with pathways of secondary metabolite biosynthesis. This will be deeply understood as we detail below for KEGG pathway mapping.

Unigenes involved in metabolic processes (18.1%) and transports (15.5%) were the two main sub-categories in the biological process category. In this category, transcripts associated to phosphorylation (10.3%), biosynthesis (7.2%), oxidation-reduction (6.5%), regulation of transcription (5.3%), response to biotic and abiotic stresses (3.1%) and signal transduction (1.0%) were also present.

**Fig 4:**
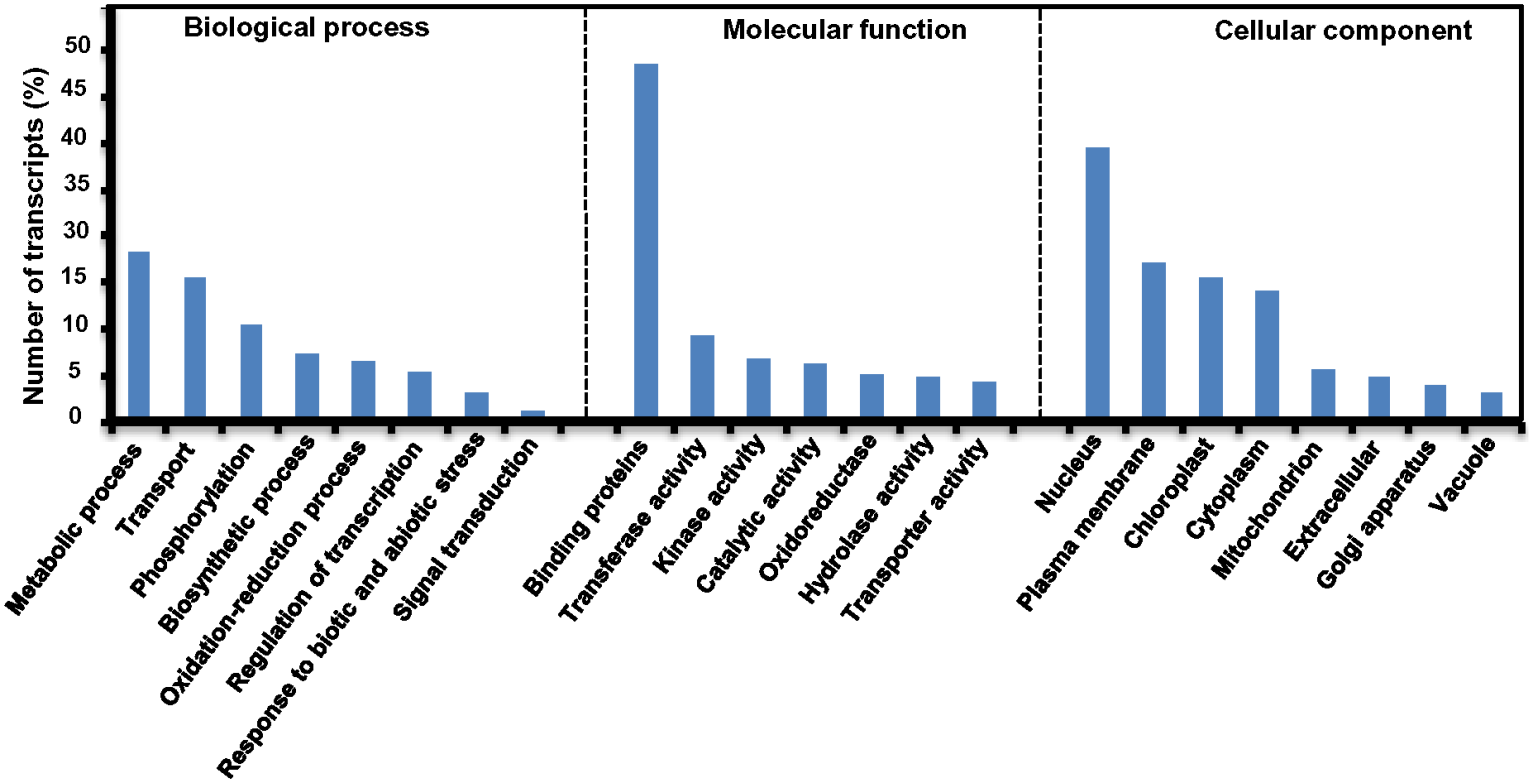
Gene Ontology classification of the assembled *Plantago ovata*transcripts in different categories of biological process, molecular function and cellular component.

Cellular components category was inhabited by maximum number of unigenes. With regard to this group, the nucleus (29.6%) and plasma membrane (17.1%) were the highly represented categories followed by chloroplast (15.3%), mitochondrion (5.4%), extracellular region (4.7%), golgi apparatus (4.0%), vacuole (2.8%), intracellular (1.1%), ribosome (0.7%), plasmodesma (0.7%), peroxisome (0.5%) and apoplast (0.2%) (Fig 4). These results however, assigned only a small percentage of the *P*. *ovata* transcripts to GO terms, possibly due to large number of uninformative gene descriptions of protein hits.

### Functional classification by KOG

**Fig 5:**
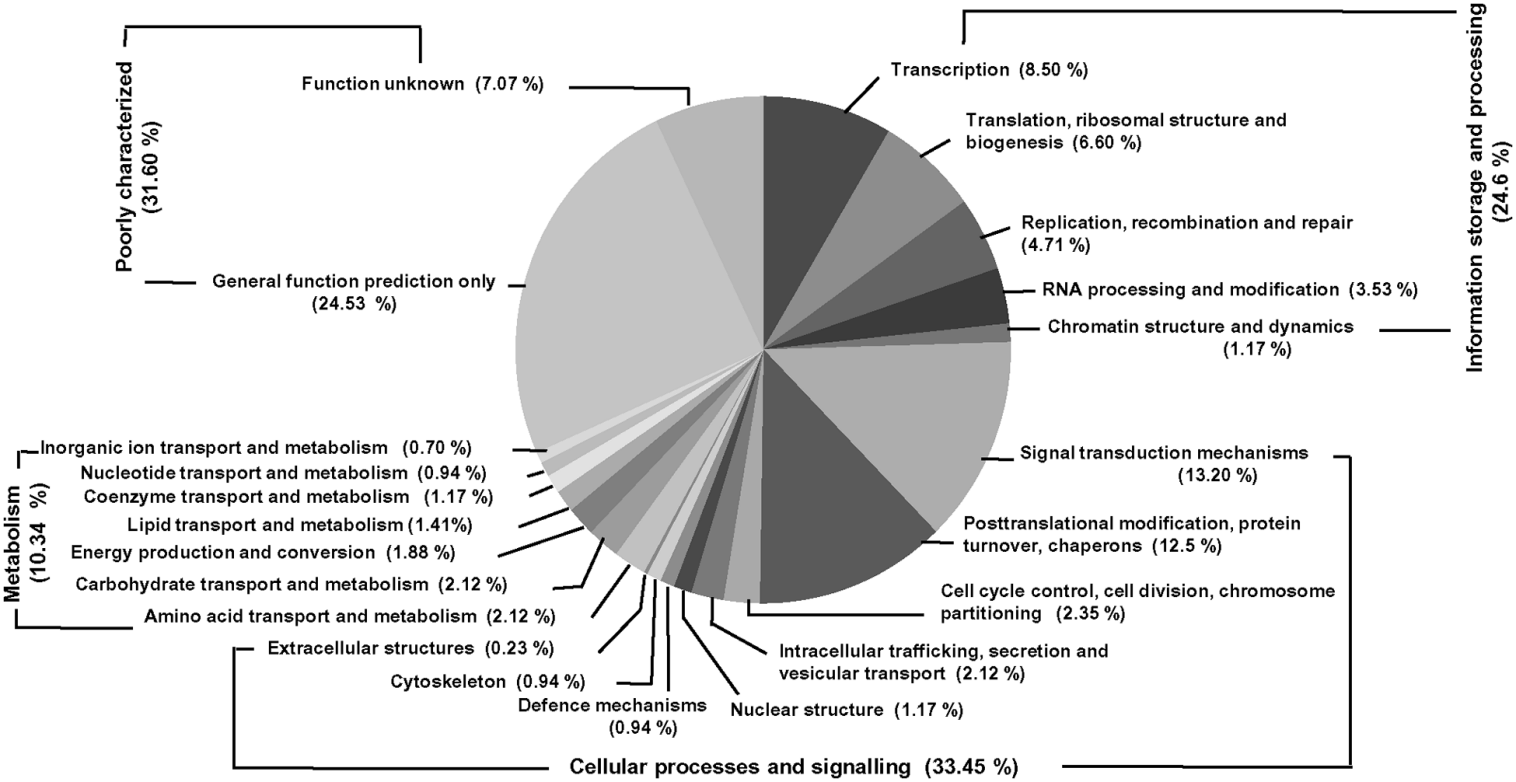
KOG functional classification of unigenes clustered into four major categories: poorly characterized, information storage and processing, cellular process and signaling and metabolism.

KOG (Eukaryotic Orthologous Groups), another form of COG (Clusters of Orthologous Groups) was used to analyze, predict and classify the transcripts with putative functions. The proteins in the COG categories were assumed to have the common ancestor protein, or to be paralogs or orthologs [32]. The transcripts were clustered into 22 categories. The largest category was general function prediction with 24.5% of transcripts. The second and third main categories were signal transduction mechanisms and post-translational modifications with 13.2% and 12.5% transcripts, respectively. Other functional categories represented were: transcription (8.4%), transcripts with unknown function (7.0%), translation, ribosome structure and biogenesis (6.6%), replication, recombination and repair (4.7%), RNA processing and modification (3.5%), and cell cycle control, cell division, chromosome partitioning (2.3%). The least represented KOG category was extracellular structures encoded by 0.2% of the unigenes (Fig 5).

### Metabolic pathways analysis through KEGG

The Kyoto Encyclopedia of Genes and Genomes (KEGG) database is a collection of manually drawn pathway maps that allows pathway-based analysis in understanding the biological functions and gene interactions [31]. To predict the metabolic pathway in *Plantago ovata*, the assembled unigenes were annotated with corresponding enzyme commission (EC) numbers in the KEGG database using KAAS (KEGG automatic annotation server) analysis tool. In the study, *Arabidopsis thaliana*, *A*. *lyrata*, *Vitis vinifera* and *Oryza sativa* japonica were used as references. The bidirectional best hit method was used to obtain KEGG orthology (KO) assignment. The output of KEGG analysis included KEGG pathways that were populated with KO assignments. A total of 1965 unigenes were classified into 319 different pathways corresponding to several KEGG modules; amongst them, metabolism pathways were the most abundant group (29.3%), with most of them involved in carbohydrate metabolism (44.4%) and amino acid metabolism (3.3%). Biosynthesis of secondary metabolites, ribosome, spliceosome were the next highly enriched categories, which were represented by 8.7%, 2.1% and 1.7% of the KEGG annotated isogenes, respectively (S4 Table).

Genes related to phenylpropanoid pathway (ko00940), terpenoid biosynthesis (ko00900), ubiquinone and other terpenoid-quinone biosynthesis (ko00130), flavonoid biosynthesis (ko00941), carotenoid biosynthesis (ko00906), sesquiterpenoid and triterpenoid biosynthesis (ko00909), monoterpenoid biosynthesis (ko00902), diterpenoid biosynthesis (ko00904), stilbenoid, diarylheptanoid and gingerol biosynthesis (ko00945), flavones and flavonol biosynthesis (ko00944) were also found. These pathways lead to the synthesis of several molecules, which have been reported to possess antioxidant activity. Several earlier studies confirm that certain members of the genus *Plantago* reveal considerable bioactivity, such as antioxidant activity [33, 34]

Many unigenes were observed to be involved in cell cycle (ko04110), plant hormone signal transduction (ko04075), fatty acid metabolism (ko01212), plant-pathogen interaction (ko04626), mRNA surveillance pathway (ko03015), photosynthesis (ko00195), biosynthesis of secondary metabolites (ko01110), DNA replication (ko03030), MAPK signaling pathway (ko04010), glycolysis/ gluconeogenesis (ko00010), RNA transport (ko03013) and Purine metabolism (ko00230). Purine metabolism is a metabolic pathway of central significance in plant growth and development [35] as purine is involved in nucleic acid synthesis. It also acts as a precursor for the synthesis of primary and secondary products [36].

Diversity of pathways and unigenes of secondary metabolites in *P*. *ovata* suggests that secondary metabolites may play important physiological functions in this plant. Some of the secondary metabolites are important plant hormones like zeatin and brassinosteroids, which play an essential role in growth and development of plants, aging and stress resistance. Future studies on the genes related to secondary metabolites will focus on adaptive evolution of this plant.

### Analysis of genes involved in metabolic pathway

#### Mucilage biosynthesis pathway genes

*P*. *ovata* is a myxospermous species like *A*. *thaliana*. The seed husk is full of mucilage, which swells on exposure to water. Being commercially and medicinally important, our focus was to identify the genes involved in mucilage biosynthetic pathway. To identify the genes that have been implicated in mucilage biosynthetic pathway, *A*. *thaliana* genome was thoroughly scanned. Of already identified genes, which are directly or indirectly involved in mucilage pathway in *Arabidopsis*, we could identify eighteen genes unequivocally in *P*. *ovata*, namely, *GATL3*, *GAUT11*, *PARVUS*, *LGT9*, *GAUT10*, *GAUT9*, *GAUT1*, *GATL6*, *GAUT4*, *GUT1*, *AP2*, *TTG1*, *MUM4*, *PRA*, *RSW3*, *MUM2*, *GL1* and *MUR4* (Table 4).

**Table 4:** Transcripts showing homology to several biosynthesis pathways: Mucilage, Carotenoid, Flavonoid and Phenylproponoid biosynthesis pathways.

S. No.	Name of the gene	Biosynthesis pathway	Transcript Information
1	Galacturonosyltransferase- like 3 (GATL3)	Mucilage biosynthesis pathway	Locus_10033_transcript_5/5
2	Galacturonosyltransferase 11 (GAUT11)		Locus_13594_transcript_1/3
3	Polygalacturonate 4- alpha- galacturonosyltransferase (PARVUS)		Locus_31653_transcript_1/1
4	Polygalacturonate 4-alpha-galacturonosyltransferase activity (LGT9)		Locus_10033_transcript_5/5
5	Galacturonosyltransferase 10 (GAUT 10)		Locus_9890_transcript_12/14
6	Galacturonosyltransferase 9 (GAUT 9)		Locus_3331_transcript_16/16
7	Galacturonosyltransferase1 (GAUT 1)		Locus_5760_transcript_1/5
8	Polygalacturonate 4-alpha-galacturonosyltransferase activity (GATL6)		Locus_15519_transcript_4/5
9	Galacturonosyltransferase4 (GAUT4)		Locus_18562_transcript_1/3
10	Glucuronoxylanglucuronosyltransferase (GUT1)		Locus_4011_transcript_6/19
11	*Arabidopsis thaliana* AP2 domain transcription factor (APETALA 2)		Locus_104366_transcript_3/8
12	Transparent Testa Glabra 1 (TTG1)		Locus_5224_transcript_1/6
13	Mucilage-Modified 4 (MUM4)		Locus_395_transcript_38/47
14	Prairie (PRA)		Locus_16030_transcript_2/6
15	Radial swelling3 (RSW3)		Locus_2050_transcript_7/12
16	Mucilage-Modified 2 (MUM2)		Locus_41504_transcript_1/1
17	Glabrous1 (GL1)		Locus_485_transcript_6/8
18	Arabinose 4-epimerase (MUR4)		Locus_28719_transcript_1/1
19	Zeta-carotene desaturase (ZDS) [EC:1.3.5.6]	Carotenoid biosynthesis pathway	Locus_27865_Transcript_2/8
20	Phytoene synthase (PSY) [EC:2.5.1.32]		Locus_5318_Transcript_5/5
21	Lycopene β-cyclase (lcyB) [EC:5.5.1.19]		Locus_15899_Transcript_3/3
22	ProlycopeneIsomerase (crtISO) [EC:5.2.1.13]		Locus_23335_Transcript_3/4
23	Carotene epsilon-monooxygenase (LUT1) [EC:1.14.99.45]		Locus_11363_Transcript_1/1
24	Zeaxanthinepoxidase (ZEP) [EC:1.14.13.90]		Locus_3049_Transcript_7/8
25	Violaxanthin de-epoxidase (VDE) [EC:1.10.99.3]		Locus_13468_Transcript_1/1
26	9-cis-epoxycarotenoid dioxygenase (NCED) [EC:1.13.11.51]		Locus_15430_Transcript_2/2
27	Abscisic-aldehyde oxidase (AAO3) [EC:1.2.3.14]		Locus_19045_Transcript_1/4
28	15-cis-zeta-carotene isomerase (Z-ISO) [EC:5.2.1.12]		Locus_20366_Transcript_2/2
29	Beta-carotene hydroxylase (chyb/crtZ) [EC:1.14.13.129]		Locus_5008_Transcript_2/2
30	Beta-ring hydroxylase (LUT5) [EC:1.14.-.-]		Locus_11330_Transcript_3/9
31	Phenylalanine ammonia lyase (PAL) [EC:4.3.1.24]	Phenylproponoid and flavonoid biosynthesis pathway	Locus_234_Transcript_30/67
32	Cinnamate 4-hydroxylase (C4H) [EC:1.14.13.11]		Locus_518_Transcript_27/37
33	4-coumarate CoA ligase (4CL 1) [EC:6.2.1.12]		Locus_7647_Transcript_1/5
34	4-coumarate CoA ligase (4CL 2) [EC:6.2.1.12]		Locus_18824_Transcript_1/1
35	4-coumarate CoA ligase (4CL 5) [EC:6.2.1.12]		Locus_107_Transcript_6/8
36	Chalcone synthase (CHS) [EC:2.3.1.74]		Locus_598_Transcript_48/50
37	ChalconeIsomerase (CHI) [EC:5.5.1.6]		Locus_7400_Transcript_15/20
38	Flavanone 3-hydroxylase (F3H) [EC:1.14.11.9]		Locus_2097_Transcript_9/16
39	Flavonoid 3’-hydroxylase (F3’H) [EC:1.14.13.21]		Locus_1209_Transcript_8/11
40	Flavonol synthase (FLS 1) [EC:1.14.11.23]		Locus_1678_Transcript_5/8
41	Flavonol synthase (FLS 2) [EC:1.14.11.23]		Locus_2087_Transcript_12/13
42	Dihydroflavonol 4-reductase (DFR) [EC:1.1.1.219]		Locus_6271_Transcript_8/10
43	Anthocyanidin synthase (ANS) [EC:1.14.11.19]		Locus_8842_Transcript_4/4
44	Anthocyanidin reductase (ANR) [EC:1.3.1.77]		Locus_14453_Transcript_1/4
45	Flavone synthase (FNS 1) [EC:1.14.11.22]		Locus_38242_Transcript_1/1
46	Flavone synthase (FNS 2) [EC:1.14.11.22]		Locus_438_Transcript_39/47
47	Flavonoid 3’-monooxygenase [EC:1.14.13.21]		Locus_1209_Transcript_8/11
48	Flavonol 3-O-methyltransferase [EC:2.1.1.76]		Locus_5520_Transcript_7/8

*AP2* (APETALA2) encodes a transcription factor of the *AP2* family. *AP2* mutants lack differentiation beyond the growth phase of both mucilage secreting cells and sub-epidermal palisade cells, suggesting that it is required for the differentiation of both outer integument derived portions of the seed coat [37]. *GAUT* (Galacturonosyltransferase) is an enzyme named as α 1, 4-D- galacturonosyltransferase. Loss in *GAUT* function results into alteration of pectin and xylan polysaccharides, as demonstrated by the altered galacturonic acid, xylose, rhamnose, galactose and arabinose composition [38]. Mutants of *GAUT11* reduced the mucilage release and lowered mucilage galacturonic acid levels, suggesting the role of *GAUT* in seed mucilage expansion and seed wall and mucilage composition [39].*MUM4* is another gene which encodes a protein of 667 amino acids. Its transcripts are apparent in all tissues but its expression gets enhanced at the time of mucilage synthesis [40]. The TRANSPARENT TESTA GLABRA1 (*TTG1*) is a WD40 repeat protein. Western et al. [41] have reported that *TTG1* is involved in the generation of mucilage in the outer layer of the seed coat. *LUH* gene has a role in seed mucilage extrusion. This seed mucilage phenotype is identical to that of *MUM2* that encodes β-galactosidase required for the modification of the mucilage. *MUM2* acts to remove the galactose/galactan branches to increase the hydrophilic properties of the mucilage, which is needed for normal hydration and expansion of the mucilage. *MUM2* mutant seed outer integument synthesizes normal amounts of mucilage but fails to extrude the mucilage upon imbibition. *LUH* and *MUM2* may act in mucilage maturation [42]. *MUR4* is another gene which leads to 50% reduction in the monosaccharide L-arabinose in most organs and affects arabinose-containing pectin cell wall polysaccharides and arabinogalactan proteins [43]. Another pleiotropic gene which affects mucilage production is RADIAL SWELLING 3 (*RSW3*). The seeds of *RSW3* mutants do not release mucilage upon hydration and have a flattened seed coat profile similar to that of *MUM4* and *TTG1* [43]. *PARVUS* gene is needed in the synthesis of pectin in plant cells. The phenotype of the plants carrying mutation in this gene supports the argument that this gene is involved in pectin synthesis [44]. *GATL* genes encode proteins involved in the cell wall biosynthesis. Data suggeststhat *GATL3*, *GATL6* and *GATL9* are involved in pectin polysaccharide synthesis, which occurs in primary wall synthesis [39]. The *LGT9* genes code for the polygalacturonate 4-alpha-galacturonosyltransferases which are involved in mucilage synthesis [45]. *GUT1* gene is needed for mucilage synthesis in plant as it encodes glucuronoxylanglucunosyltransferase [45]. In addition, several other genes were also identified which are also required for synthesis of mucilage components but their direct role in mucilage pathway is yet to be confirmed. *PRA* is one such gene whose function is yet unknown but its mutant shows reduced mucilage as compared to wild type plants [44] (Table 4).

#### Carotenoid biosynthetic pathway

Several unigenes were annotated as encoding enzymes involved in carotenoid synthesis based on the KEGG pathway assignments. The biochemical pathway starts with the synthesis of phytoene and proceeds along a single path to lycopene. Phytoene synthase (EC 2.5.1.32, 1 unigene) catalyses the condensation of two molecules of geranylgeranyl pyrophosphate to produce 15-cis phytoene. Then, a set of reactions, all trans-lycopenes, are produced from phytoene. Amongst the four genes required in pathway, three were identified from the present data; *15-cis-zeta-carotene isomerase* (*Z*-*ISO*) (1 unigene), *zeta-carotene desaturase* (*ZDS*) (EC 1.3.5.6, 1 unigene) and *carotene isomerase* (*crtISO*) (EC 5.2.1.13, 1 unigene). Lycopene is the branching point of this pathway, beyond which carotenoid biosynthesis bifurcates under the catalysis of lycopene cyclases to produce epsilon- and beta-carotenoids, which are cyclic precursors of xanthophylls. Identification of transcripts coding for *carotene epsilon-monooxygenase LUT1* (EC 1.14.99.45, 1 unigene), *carotene beta-ring hydroxylase LUT5* (EC 1.14.99.-, 1 unigene) and identification of one gene as a promising candidate *lcyB* (EC 5.5.1.19, 1 unigene) point towards the production of both lutein and zeaxanthin in *P*. *ovata*. *LUT1* also known as *LUTEIN DEFICIENT 1* possesses epsilon hydroxylase activity and *LUT5* is a cytochrome P450-type monooxygenase that possesses β-ring hydroxylase activity [46]. The presence of *beta-carotene 3-hydroxylase chyb/crtZ* (EC 1.14.13.129) indicates chanelling of the pathway towards the production of zeaxanthin. Further, epoxidation of zeaxanthin by *zeaxanthinepoxidase ZEP*) (EC 1.14.13.90, 1 unigene) produces violaxanthin. This reaction is reversed by *Violaxanthin de-epoxidase* (*VDE*) (EC 1.10.99.3, 1 unigene) to give rise to the xanthophyll cycle for plants to adapt to high light stress [47]. Apart from these, presence of transcripts corresponding to genes *NCED* (EC 1.13.11.51, 1 unigene) and *abscisic-aldehyde oxidase AAO3* (EC 1.2.3.14) demonstrates the presence of abscisic acid biosynthesis. Abscisic acid (ABA) is a plant hormone involved in seed development and germination and involved in responses to various environmental stresses [48] (Table 4).

#### Flavonoid biosynthesis pathway

Genes associated with flavonoid biosynthesis pathway were also identified in our dataset. Flavonoids are synthesized via the phenylpropanoid pathway and are converted from phenylalanine to chalcone by the enzymes phenylalanine ammonia lyase (EC 4.3.1.24, 1 unigene), cinnamate 4-hydroxylase (EC 1.14.13.11, 1 unigene), 4-coumarate CoA ligase (EC 6.2.1.12, 3 unigenes) and chalcone synthase (EC 2.3.1.74, 1 unigene). Chalcone isomerase (EC 5.5.1.6, 1 unigene) catalyses the isomerisation of chalcones into naringenin. Naringenin can be converted by flavonoid 3’-hydroxylase (EC 1.14.13.21, 1 unigene) to produce eriodictyol. Flavone synthase (EC 1.4.11.22, 2 unigenes) catalyses the conversion of flavanones to flavones and flavanone 3-hydroxylase (EC 1.14.11.9, 1 unigene) can convert these flavanones to dihydroflavonols. Dihydroflavonols can then lead to the production of flavonols and flavan-3,4-diols (leucoanthocyanidin), reactions being catalysed by flavonol synthase (EC 1.14.11.23, 2 unigenes) and by dihydroflavonol4-reductase (EC 1.1.1.219, 1 unigene) respectively. Leucoanthocyanidins can be converted either to anthocyanidins and subsequently anthocyanins through the subsequent action of anthocyanidin synthase (EC 1.14.11.19, 1 unigene) or reduced to catechins through the action of enzyme anthocyanidin reductase (EC 1.3.1.77, 1 unigene). The description showed vertical pathway responsible for the formation and conversion of the sub-categories of Flavonoids (Table 4).

Genes of phenylpropanoid biosynthesis pathway have been reported in several plant species such as *Camellia sinensis*, *Zea mays*, *Arabidopsis thaliana*, *Vitis vinifera* [49], to name a few. Presence of transcripts coding for the enzymes flavone synthase (FS) and flavonol sythase (FLS) strongly suggests that the two classes of flavonoids (flavones and flavonols) are present in *Plantago ovata*. This finding is in agreement with the studies of Kawashty et al. [50], Beara et al. [33] and Jankovic et al. [51] wherein flavones and flavonols have been reported to be major flavonoids present in *Plantago* species.

The absence of F3’5’H and LAR and the presence of F3’H, DFR and ANS points towards the formation of proanthocyanidins via ANS/ANR branch of the pathwayleading to the synthesis of cyanidin and pelargonidin based pigments, which impart brick red to orange coloration. The colour of the mature ovary/ fruit of *P*. *ovata* may be the result of the presence and expression of these genes, as it is known that proanthocyanidins (also known as condensed tannins) are responsible for seed coat pigmentation and may function as a barrier to fungal infection of embryos [52]. The chemopreventive and chemotherapeutic properties of the seed husk may be because of the antioxidant and anti-inflammatory properties of the proanthocyanidins [52].

### Identification of transcription factors

Transcription factors, represented by multigene families are key regulatory factors. These bind to specific DNA sequences and are involved in regulation of gene expression. They may be considered as molecular switches that link signal transduction pathways to gene expression. These are highly conserved in eukaryotes, especially plants and ~ 7% of all genes encode for transcription factors [17, 53, 54].

**Fig 6:**
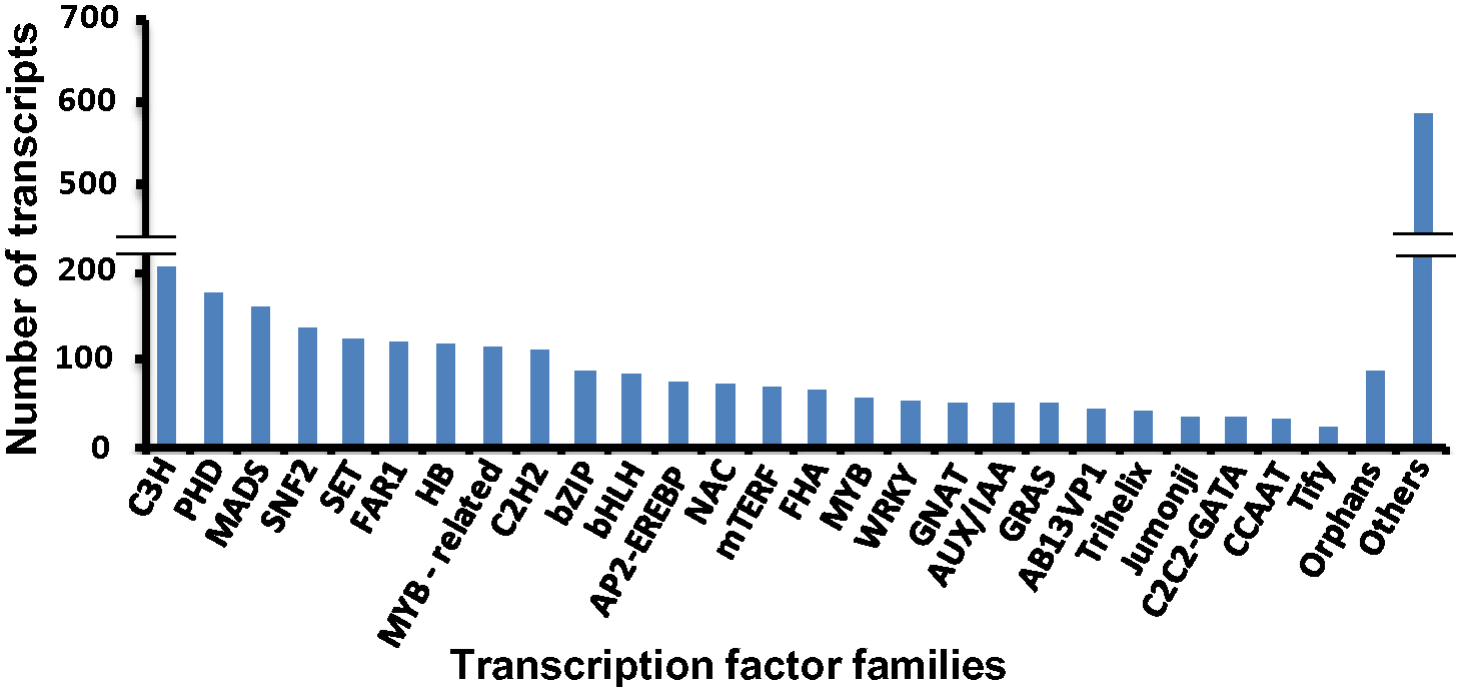
Distribution of *Plantago ovata* transcripts in different transcription factor families. C3H, PHD and MADS transcription factor families were enriched in number however CCAAT and Tify were least in number.

*P*. *ovata* transcripts were searched against PlantTFDB using BLASTX to identify the putative transcription factors. A total of 2,849 transcripts (with an e value ≤1e-10) matched in TFDB corresponding to 78 TF families and represent 6.06% of *P*. *ovata* total transcripts (S3 Table). Most abundant TF family was C3H (7.3%), followed by PHD (6.2%), MADS (5.6%), SNF2 (4.7%), SET (4.3%), FAR1 (4.2%), HB (4.0%),C2H2 (3.93%), MYB-related (3.96%), bZIP (3.0%), bHLH (2.9%), NAC (2.6%), FHA (2.3%) and where as Alfin-like, BBR/BPC, MBF1 (0.1% each); MED7, VOZ, PBF-2-like, TIG, MED6, S1Fa-like (0.07% each) and SOH1 (0.03%) were the least abundant TF families (Fig 6). Total list of TF are also provided in S5 Table.

Several metabolic pathways in plants are subjected to transcriptional regulation through various transcription factors. In the present investigation, genes encoding TFs were mapped on KEGG pathways to discover the transcription factor regulatory pathways. Various secondary metabolic pathways leading to the synthesis of mucilage and secondary metabolites like, phenylpropanoid, terpenoid, carotenoid and flavonoid pathways have been reported to be operational in *P*. *ovata* [2, 51]. Therefore, in order to enhance the production of these metabolites, it is necessary to identify the regulatory molecules controlling these pathways so that strategies for increasing their amount can be devised.

Members of MYB family play regulatory roles in some important metabolic pathways. With their role in regulation of mucilage biosynthesis, MYB61 mutants have been found to be deficient in seed mucilage extrusion upon imbibition [55]. MYB TFs have also been reported to regulate epidermal cell fate and seed coat development in *Arabidopsis*. This family of transcription factors has been primarily involved in governing the flavonoid biosynthesis pathway [36] and flavonol biosynthesis in *Arabidopsis* [56, 57]. They associate with TFs bHLH and WD40 and the resulting MBW complex regulates the later steps of flavonoid biosynthesis, particularly the ones leading to the synthesis of anthocyanins and condensed tannins [58, 59]. Weisshaar and Jenkins [60] have also reported bZIP and bHLH TFs to play a regulatory role in flavonoid and anthocyanin biosynthesis. Myb related TFs families have been associated with the phenylpropanoid biosynthesis and anthocyanin biosynthesis pathways [60].

Nieuwenhuizen et al. [61] have documented the importance of NAC TFs in controlling monoterpene production in kiwifruit. TF families AP2, WRKY have also been reported to be involved in regulation of terpenoid pathways [62]. Plant ZEP (zeaxantihinepoxidase) protein (xanthophyll cycle; carotenoid biosynthesis) has a requirement for phosphopeptide binding domain (forkhead associate domain or FHA domain), which has been reported to be involved in protein-protein interaction [63]. Similarly, members of AP2/EREBP family also play a role in regulation of carotenoid biosynthesis [64]. Genes of mucilage biosynthesis pathway are also regulated by AP2 class of TF in *Arabidopsis* [37].

Regulation of several important cell functions and gene expression can be modified by taming and improvising the interaction of various transcription factors with nucleic acids and proteins. This might aid in altering the regulatory steps of various important metabolic pathways that can help to increase the medicinal properties of *P*. *ovata*, therefore, enhancing its importance.

### Identification of SSRs

Microsatellites are tandem repeats of DNA sequences of only a few base pairs (1–6 bp) in length. These markers are reproducible, multiallelic in nature, show co-dominant inheritance, are relatively abundant in the genome and have good genome coverage [65]. These properties make them useful in several applications in plant genetics and breeding like genome mapping, gene tagging, cultivar identification, estimation of genetic relatedness and germplasm conservation [66]. We have recently reported the cross-genus amplification of several SSR markers (based on genus *Malus* and *Phaseolus*) in several accessions of *P*.*ovata* and different species of the genus *Plantago* [67].

*P*. *ovata* transcripts were searched with perl script MISA for the identification of SSRs, which resulted in 1,224 SSRs in 1,119 (2.3%) unigenes. The number of transcripts containing more than one SSR was 95. It was observed that 70 SSRs are present in compound formation wherein; the maximal number of bases interrupting two SSRs was 100 (Table 5) (S6 Table).

**Table 5:** Statistics of SSRs identified in *Plantago* transcripts.

SSR Mining	
Total number of sequences examined	46,955
Total size of examined sequences (bp)	19,251,438
Total number of identified SSRs	1,224
Number of SSRs containing sequences	1,119
Number of sequences containing more than one SSR	95
Number of SSRs present in compound formation	70
Maximal number of bases interrupting two SSRs in a compound microsatellite	100

**Fig 7:**
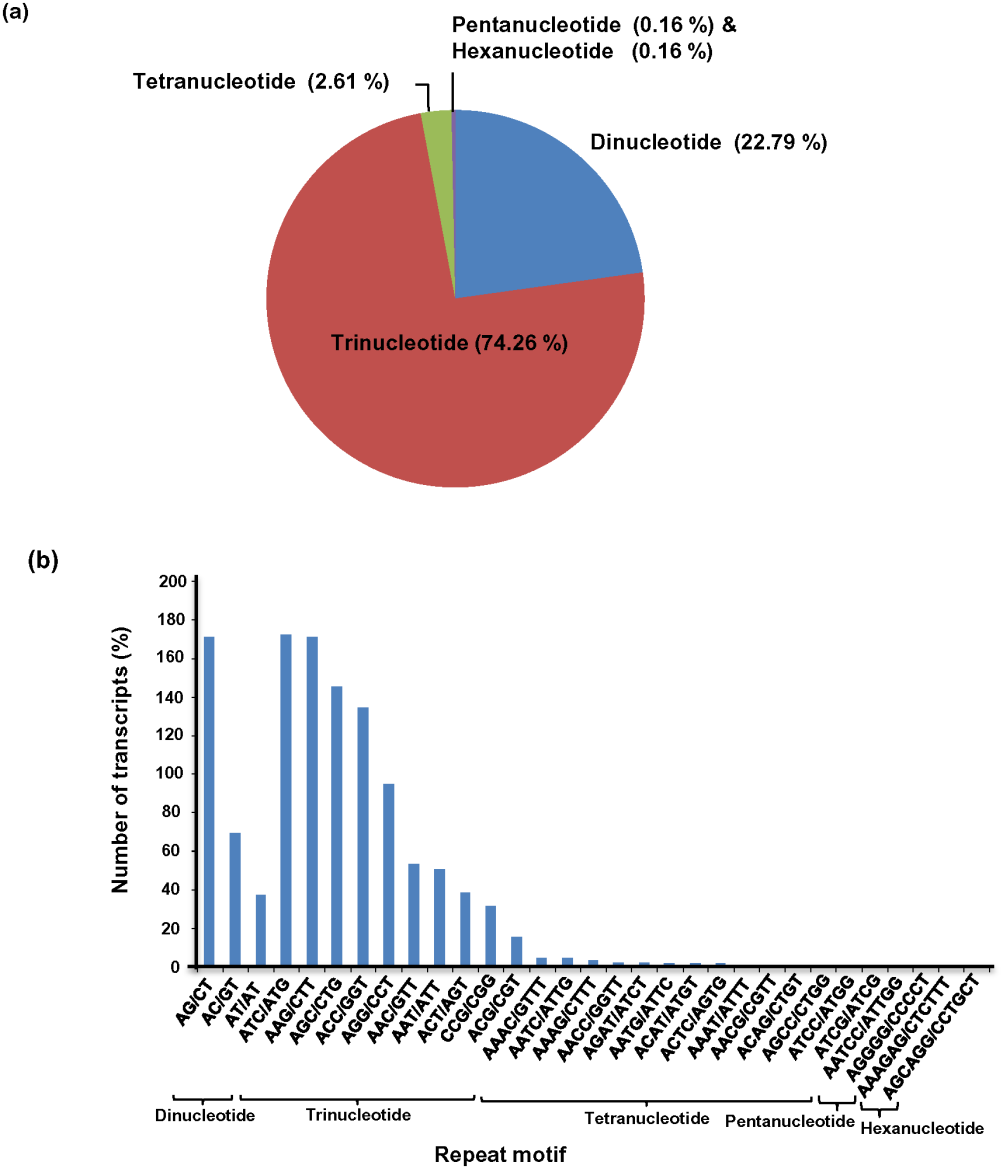
Distribution of SSR repeat type and repeat motifs. Fig (a) different repeat type SSRs and Fig (b) different repeat motifs and percentage of transcripts corresponding to them.

Analysis of repeat type SSRs depicted that tri-nucleotide SSRs represented the largest fraction (74.2%), followed by di-nucleotide (22.7%) SSRs, as also reported in several studies in other plants [17, 21, 29, 68, 69]. Tetra-nucleotide (2.6%) SSRs were next highly represented class. Only a small fraction of penta- and hexa-nucleotide SSRs (0.1% each) were identified in *P*. *ovata* unigenes with same frequencies of the repeat units (Fig 7a). The most abundant motifs of tri-nucleotide repeat units were ATC/ATG and AAG/CTT with frequencies of 18.9% and 18.8% SSRs, respectively. Among the di-nucleotide repeat units, AG/CT and AC/GT type SSRs were the most abundant with the frequencies of 61.2% and 25.0%, respectively (Fig 7b).

The genic SSRs identified in the current study will add to the already existing repertoire of microsatellites in *P*. *ovata*. Apart from outlining the variation in transcribed and known functional genes, their presence in the transcripts of geneshint towards their role in gene expression and function. Also, they can highlight any non-random association between marker, genes or QTLs in a population and can be used to create linkage map/ linkage population. The current study reports genome-wide SSRs for the first time in *P*. *ovata*. This can further aid in marker-assisted selection to speed up the conventional plant breeding.

### Identification of noncoding RNAs (ncRNAs)

**Fig 8:**
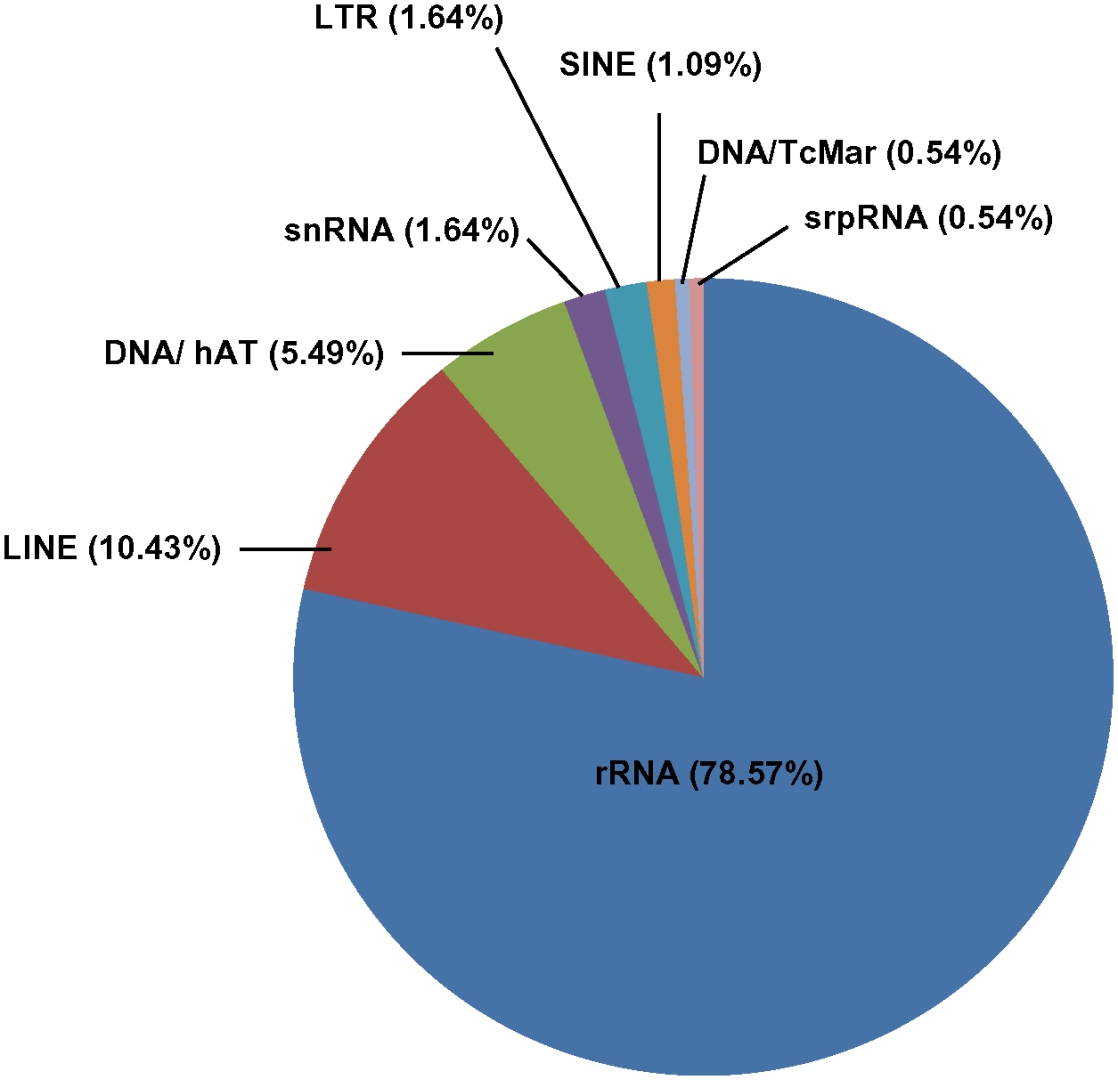
Different classes of non-coding RNAs (ncRNAs) as identified by the Repeat Masker software in *P*. *ovata*.

The advent of new sequencing technologies has provided an insight into identification of new non-coding RNA (ncRNA) classes such as promoter-associated RNAs and long RNAs [70]. Noncoding RNA (ncRNA) produces a set of transcripts that function directly as structural, catalytic or regulatory RNAs rather than expressing mRNAs. They are known to fulfill central functions in the cell including control of chromosome dynamics, RNA editing, splicing, RNA regulation and mRNA destruction. They have also been observed to play important role in stress responses [71, 72]. During the present study all the unigenes were analyzed by Repeat masker, resulting in identification of a total of 182 non coding RNAs belonging to various repeat families. It was observed that 78.57% of the total ncRNAs belonged to the repeat class/ family rRNA followed by LINE (10.43%), DNA/hAT (5.49%) whereas, DNA/TcMar and srpRNA were the least represented families with the frequency of 0.54% each (Fig 8).

Non-coding RNAs play an important role in regulation of gene expression and important cellular functions like protein synthesis (rRNA) [73]; RNA transcription and post transcriptional regulation including splicing, translation (Long ncRNA e.g. LINE, SINE) [74]; in protein trafficking and their sorting within the cells (srpRNA) [75], to name a few. Targeting these ncRNAs can help control and regulate some of the important cell processes and functions, which can further aid in improving the importance (medicinal and economical) of the plant under study.

### Quantitative real-time PCR (qRT-PCR) analysis

Quantitative real-time PCR (qRT-PCR) analysis was undertaken in *P*. *ovata* and compared with *A*. *thaliana*, with regard to 8 differentially expressed genes namely *PARVUS*, *GUT1*, *PRA*, *MUR4*, *GL1*, *GAUT9*, *GAUT1* and *GAUT4*. The expression of these genes was analyzed in different tissues of *P*. *ovata* (leaf, root and spike) and *A*. *thaliana* (leaf, root and flower). Transcripts of these genes were detected in the tissues at variable amounts (S6 Fig). In both the plants, the expression levels in the different tissues were compared with expression in leaves, latter chosen as reference tissues. The results showed that the expression patterns of transcripts in *Arabidopsis* were mostly in compliance with what has already been reported by other workers [39, 76, 77, 78, 79]. However, the expression levels of some genes in *P*. *ovata* and *A*. *thaliana* vary. For example, *PARVUS*, *GUT1*, *PRA* and *GL1* showed higher expression in spikes of *P*. *ovata* whereas in flowers of *A*. *thaliana*, *GAUT9*, *GAUT1* and *GAUT4* showed higher expression. The expression of *MUR4* was observed to be higher in roots as compared to flowers/spikes. This can be explained on the basis of the fact that mucilage production is a stress response and the root, particularly root tip represents the first organ in perceiving the water stress [80]. Overall, the expression study does validate the results obtained with regard to transcriptome study of some of the genes involved in mucilage biosynthetic pathway. However, detailed investigations on identification of specific genes involved in the pathway need to be undertaken.

## Conclusions

In this study, a comprehensive database has been prepared to manage and explore the EST collection from ovaries of the *Plantago ovata*. RNA-seq was used to obtain short—read sequence data of this commercially and medicinally important plant. *Denovo* assembly approach was used to assemble 31,280,458 PE sequence reads to generate 46,955 unigenes with an average sequence length of 410 bp. A total of 61.6% unigenes were functionally annotated and were found to be involved in different biological processes. KEGG pathway mapping provided an insight towards several important pathways in plant, including various secondary metabolic pathways like mucilage biosynthesis, flavanoid and carotenoid biosynthesis pathways. In addition, various genes involved in different pathways leading to the formation of several antioxidants were also identified. To aid and accelerate future genome-wide study in this plant, assignment of GC content, prediction of several conserved transcription factor families and functional categories by GO annotation and KOG classification was also carried out. The ncRNAs identified in thetranscriptome paves a way for the clear understanding of several processes, including important cell functions and regulation of gene expression. However, the functional genomics of ncRNAs will be a daunting task to intersect and modulate the complex gene activity mechanisms. The genomic-SSRs identified in this studyrepresent the first report of its kind, which will provide a very good resource and cost effective option to develop functional markers for marker assisted breeding and will also help in the genetic improvement of medicinally important plant.

## Supporting Information

S1 Fig Relative size of ovaries at different developmental stages of seed. (PDF)

S2 Fig Pie chart showing QC summary depicting the percentage of high quality, low quality and contaminated reads. (PDF)

S3 Fig Phred quality score distribution of read sequences before and after filtering low—quality reads and reads containing adaptor/primer sequences. Fig (a) Phred quality score distribution of forward read in paired end library and Fig (b) Phred quality score distribution of reverse reads in paired end library.
(PDF)

S4 Fig Comparison of *de novo* assembly obtained through (a) Velvet, (b) ABySS and (c) Oases programs. The bars indicate number of transcripts (left axis). The lines indicate N50 length (triangles) and average transcript length (rectangles) in bp (right axis).
(PDF)

S5 Fig Sequence length distribution of the assembled transcripts. Most of the transcripts fall into 100–200 bp length whereas numbers of transcripts between 1401–1500 bp are less. Transcripts with length >1500 bp is highest in number as compared to transcripts with length 1100 to 1500 bp.
(PDF)

S6 Fig Expression pattern of eight transcripts using qRT-PCR in different tissues of *P*. *ovata* and *A*. *thaliana*. PS-*Plantago* Spike, AF-*Arabidopsis* Flower, PR-*Plantago* Root, AR-*Arabidopsis* Root, PL-*Plantago* Leaf and AL-*Arabidopsis* Leaf. Y-axis represents Relative quantification (R.Q.) values as compared to reference tissue (leaf).
(PDF)

S1 Table Time line followed during the development of the seed in *Plantago ovata*. (DOC)

S2 Table Efficiency of the primers used in qPCR for studying comparative expression. (DOC)

S3 Table Assembly statistics of *P*. *ovata* transcripts using different assemblers. (XLS)

S4 Table Table enlisting all the *Plantago ovata* transcripts that were annotated with KEGG ID's and the information pertaining to that ID is also mentioned. (XLS)

S5 Table Table enlisting the number of *P*. *ovata* genes/transcripts coding for various transcription factor families. (XLS)

S6 Table Table enlisting the number and type of all the SSRs identified in *Plantago ovata* transcriptome. (XLS)
