# Supplementary figures and images for: De Novo Transcriptome Analysis of Medicinally Important Plantago ovata Using RNA-Seq
Source: PLoS One. 2016 Mar 4;11(3):e0150273. doi: 10.1371/journal.pone.0150273 (PMC4778938; doi:10.1371/journal.pone.0150273)

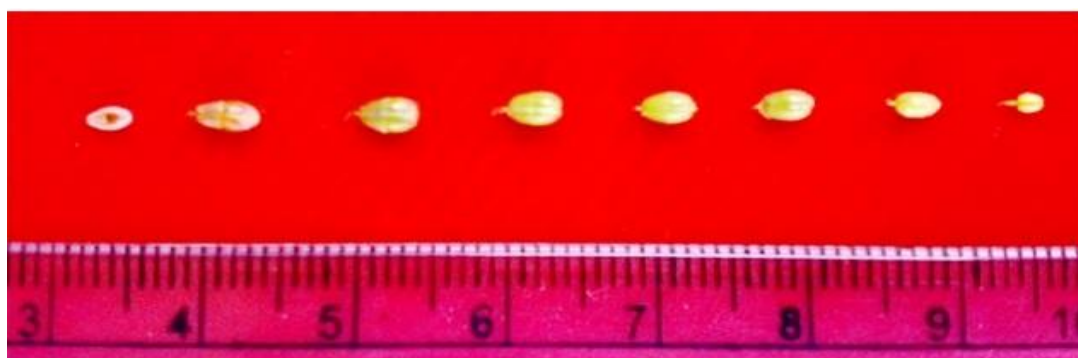

**S1 Fig. Relative size of ovaries at different developmental stages of seed.**

Supplement: S1 Fig — (PDF) [file pone.0150273.s001.pdf]
