# Supplementary material for: De Novo Transcriptome Analysis of Medicinally Important Plantago ovata Using RNA-Seq
Source: PLoS One. 2016 Mar 4;11(3):e0150273. doi: 10.1371/journal.pone.0150273 (PMC4778938; doi:10.1371/journal.pone.0150273)
Supplement: S2 Fig — (PDF) [file pone.0150273.s002.pdf]

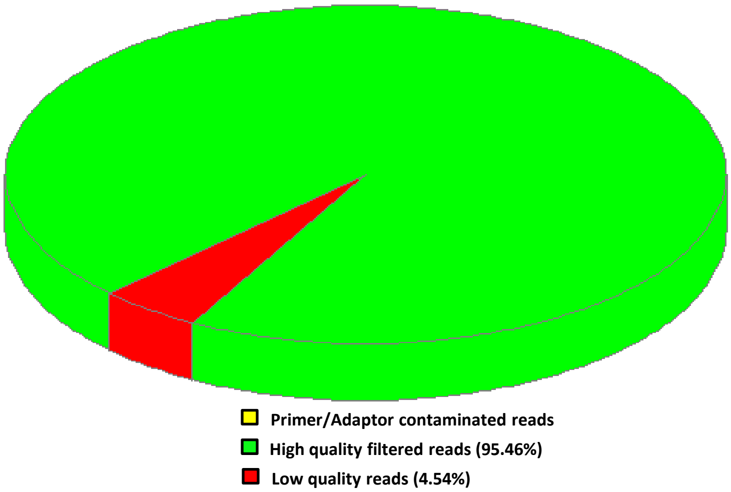

S2 Fig. Pie chart showing QC summary depicting the percentage of high quality, low quality and contaminated reads.
