# Supplementary material for: De Novo Transcriptome Analysis of Medicinally Important Plantago ovata Using RNA-Seq
Source: PLoS One. 2016 Mar 4;11(3):e0150273. doi: 10.1371/journal.pone.0150273 (PMC4778938; doi:10.1371/journal.pone.0150273)
Supplement: S3 Fig — Fig (a) Phred quality score distribution of forward read in paired end library and Fig (b) Phred quality score distribution of reverse reads in paired end library. (PDF) [file pone.0150273.s003.pdf]

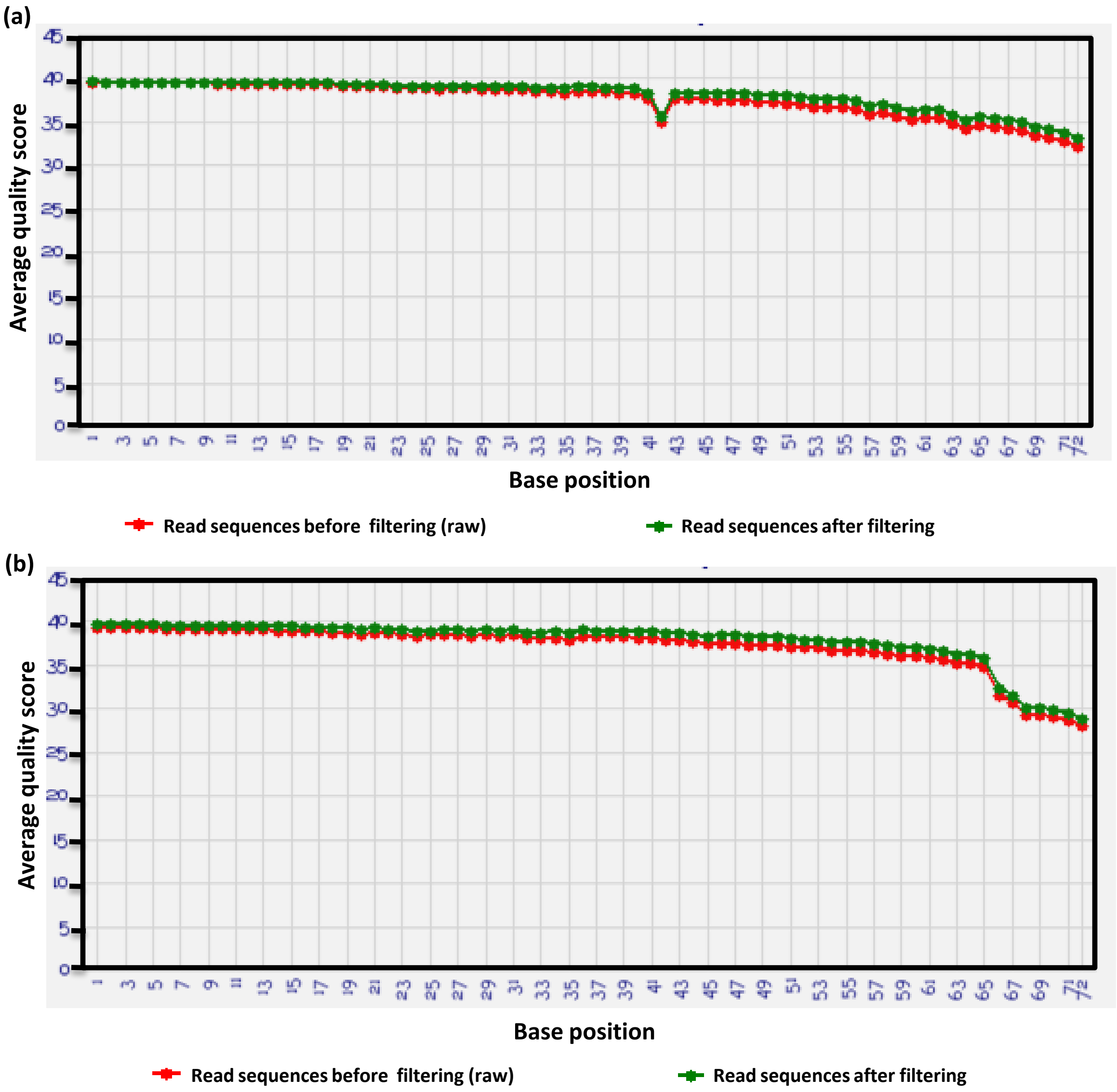

S3 Fig. Phred quality score distribution of read sequences before and after filtering low – quality reads and reads containing adaptor/primer sequences. Fig. (a) Phred quality score distribution of forward read in paired end library and fig. (b) Phred quality score distribution of reverse reads in paired end library.
