# Supplementary material for: De Novo Transcriptome Analysis of Medicinally Important Plantago ovata Using RNA-Seq
Source: PLoS One. 2016 Mar 4;11(3):e0150273. doi: 10.1371/journal.pone.0150273 (PMC4778938; doi:10.1371/journal.pone.0150273)
Supplement: S5 Fig — Most of the transcripts fall into 100–200 bp length whereas numbers of transcripts between 1401–1500 bp are less. Transcripts with length >1500 bp is highest in number as compared to transcripts with length 1100 to 1500 bp. (PDF) [file pone.0150273.s005.pdf]

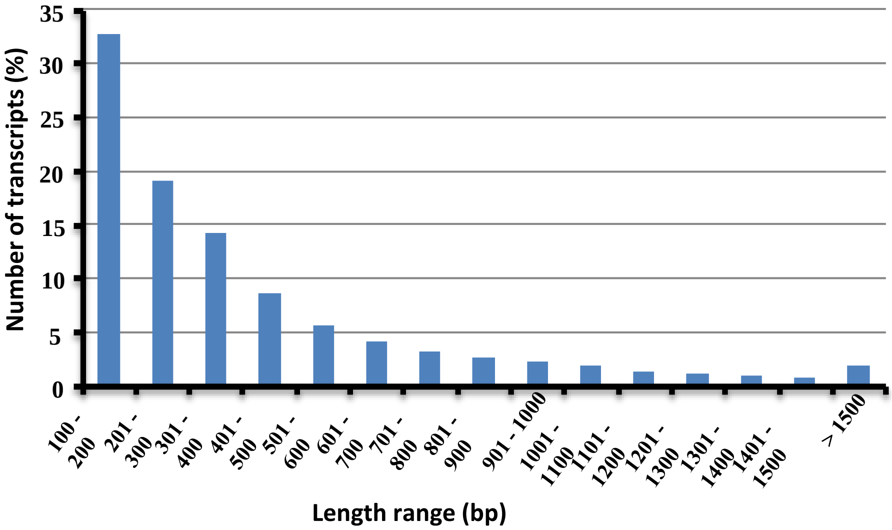

S5 Fig. Sequence length distribution of the assembled transcripts. Most of the transcripts fall into 100-200 bp length whereas number of transcripts between 1,401-1,500 bp are less. Transcripts with length >1,500 bp is highest in number as compared to transcripts with length 1,100 to 1,500 bp.
