# Supplementary material for: De Novo Transcriptome Analysis of Medicinally Important Plantago ovata Using RNA-Seq
Source: PLoS One. 2016 Mar 4;11(3):e0150273. doi: 10.1371/journal.pone.0150273 (PMC4778938; doi:10.1371/journal.pone.0150273)
Supplement: S6 Fig — PS-Plantago Spike, AF-Arabidopsis Flower, PR-Plantago Root, AR-Arabidopsis Root, PL-Plantago Leaf and AL-Arabidopsis Leaf. Y-axis represents Relative quantification (R.Q.) values as compared to reference tissue (leaf). (PDF) [file pone.0150273.s006.pdf]

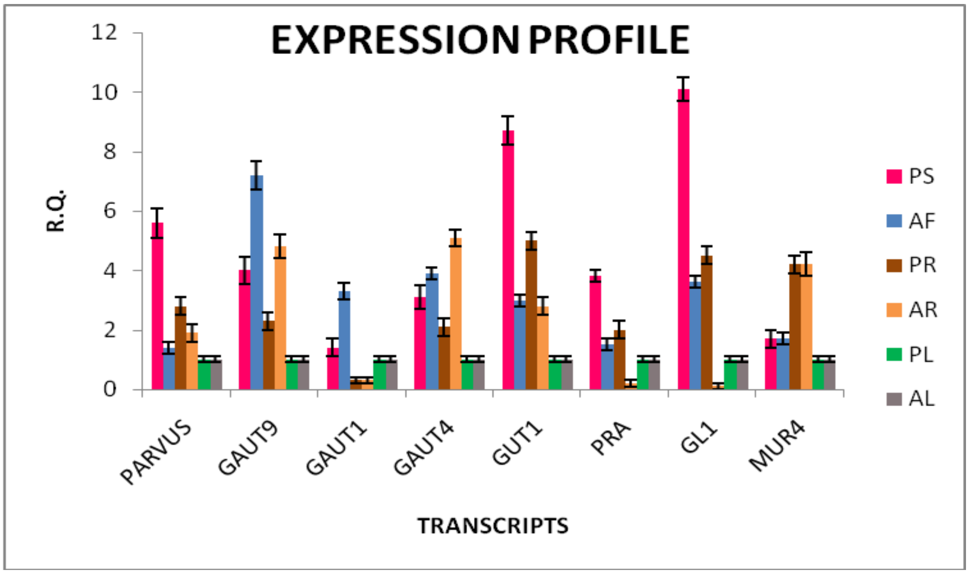

S6 Fig. Expression pattern of eight transcripts using qRT-PCR in different tissues of *P. ovata* and *A. thaliana*. PS-*Plantago* Spike, AF-*Arabidopsis* Flower, PR-*Plantago* Root, AR-*Arabidopsis* Root, PL-*Plantago* Leaf and AL-*Arabidopsis* Leaf. Y-axis represents Relative quantification (R.Q.) values as compared to reference tissue (leaf).
