# Supplementary material for: De Novo Transcriptome Analysis of Medicinally Important Plantago ovata Using RNA-Seq
Source: PLoS One. 2016 Mar 4;11(3):e0150273. doi: 10.1371/journal.pone.0150273 (PMC4778938; doi:10.1371/journal.pone.0150273)
Supplement: S1 Table — (DOC) [file pone.0150273.s007.doc]

**S1 Table. Time line followed during the development of the seed in *Plantago ovata***

| **Days after pollination (DAP)** | **Size of the ovule (mm) ± S.D.** | **Phenotypic observations** |
| --- | --- | --- |
| 0 | 0.6±0.03 | Soft, fragile, green |
| 1 | 0.7±0.02 | Soft, fragile, green |
| 2 | 0.9±0.02 | Soft, green |
| 3 | 1.3±0.03 | Soft, green |
| 4 | 1.7±0.02 | Soft, green |
| 5 | 2.0±0.19 | Soft, green |
| 6 | 2.4±0.17 | Soft, green |
| 7 | 3.0±0.17 | Soft, green |
| 10 | 3.5±0.19 | Soft, unripe, green |
| 15 | 3.7±0.13 | Soft, unripe, green |
| 20 | 4.0±0.18 | Hard, half mature, purplish ovules |
| 25 | 4.0±0.17 | Hard, immature, purplish ovules bearing seeds |
