# Supplementary material for: De Novo Transcriptome Analysis of Medicinally Important Plantago ovata Using RNA-Seq
Source: PLoS One. 2016 Mar 4;11(3):e0150273. doi: 10.1371/journal.pone.0150273 (PMC4778938; doi:10.1371/journal.pone.0150273)
Supplement: S2 Table — (DOC) [file pone.0150273.s008.doc]

**S2 Table**. Efficiency of the primers used in qPCR for studying comparative expression

| **Gene** | **Primer code** | **Primer sequence** |
| --- | --- | --- |
| ***PARVUS*** | MGP1-F | 5’TCGCTCTCGCTCACGTTTG3’ |
|  | MGP1-R | 5’ACCATTACCCCCGTGTTGAA3’ |
| ***GAUT9*** | MGP2-F | 5’TGGCGCTCACATCGAAGTT3’ |
|  | MGP2-R | 5’TGGGACATAAGAAGAGTTGAGAAATTT3’ |
| ***GAUT1*** | MGP3-F | 5’CTTTGGTGCAATGAATATGTGGTT3’ |
|  | MGP3-R | 5’TTCGACGTTGATGGTAGCTTTC3’ |
| ***GAUT4*** | MGP4-F | 5’ACTGCTTTGGAAACTCGGAACT3’ |
|  | MGP4-R | 5’GTGTAAGTGCGATTCCAGAAGGT3’ |
| ***GUT1*** | MGP5-F | 5’CGTGATGAGGAGCGCTGTAA3’ |
|  | MGP5-R | 5’CCCCTCAGTTCTGTTCCAATATG3’ |
| ***PRA*** | MGP6-F | 5’TCATGGCACCAATGTCAGTCA3’ |
|  | MGP6-R | 5’TTCGAGTGGGCCTGCAA3’ |
| ***GL1*** | MGP7-F | 5’GAGAGAGATTCCAGACCCAGTTAAA3’ |
|  | MGP7-R | 5’ACTCAGCCAATCCCAAATTCA3’ |
| ***MUR4*** | MGP8-F | 5’GGGATTACATCCATGTCACTGATC3’ |
|  | MGP8-R | 5’GCTCTGGTTTTGCCTTTTGAA3’ |
| ***ACTIN*** | ACTIN-F | 5’AGGTATTGTGTTGGACTCTGGTGAT3’ |
|  | ACTIN-R | 5’ACGGAGAATGGCATGTGGAA3’ |

| **Gene** | **Primer code** | **Primer sequence** |  |  |
| --- | --- | --- | --- | --- |
| ***PARVUS*** | MGP1-F | 5’TCGCTCTCGCTCACGTTTG3’ |  |  |
|  | MGP1-R | 5’ACCATTACCCCCGTGTTGAA3’ |  |  |
| ***GAUT9*** | MGP2-F | 5’TGGCGCTCACATCGAAGTT3’ |  |  |
|  | MGP2-R | 5’TGGGACATAAGAAGAGTTGAGAAATTT3’ |  |  |
| ***GAUT1*** | MGP3-F | 5’CTTTGGTGCAATGAATATGTGGTT3’ |  |  |
|  | MGP3-R | 5’TTCGACGTTGATGGTAGCTTTC3’ |  |  |
| ***GAUT4*** | MGP4-F | 5’ACTGCTTTGGAAACTCGGAACT3’ |  |  |
|  | MGP4-R | 5’GTGTAAGTGCGATTCCAGAAGGT3’ |  |  |
| ***GUT1*** | MGP5-F | 5’CGTGATGAGGAGCGCTGTAA3’ |  |  |
|  | MGP5-R | 5’CCCCTCAGTTCTGTTCCAATATG3’ |  |  |
| ***PRA*** | MGP6-F | 5’TCATGGCACCAATGTCAGTCA3’ |  |  |
|  | MGP6-R | 5’TTCGAGTGGGCCTGCAA3’ |  |  |
| ***GL1*** | MGP7-F | 5’GAGAGAGATTCCAGACCCAGTTAAA3’ |  |  |
|  | MGP7-R | 5’ACTCAGCCAATCCCAAATTCA3’ |  |  |
| ***MUR4*** | MGP8-F | 5’GGGATTACATCCATGTCACTGATC3’ |  |  |
|  | MGP8-R | 5’GCTCTGGTTTTGCCTTTTGAA3’ |  |  |
| ***ACTIN*** | ACTIN-F | 5’AGGTATTGTGTTGGACTCTGGTGAT3’ |  |  |
|  | ACTIN-R | 5’ACGGAGAATGGCATGTGGAA3’ |  |  |
