# Supplementary material for: De Novo Transcriptome Analysis of Medicinally Important Plantago ovata Using RNA-Seq
Source: PLoS One. 2016 Mar 4;11(3):e0150273. doi: 10.1371/journal.pone.0150273 (PMC4778938; doi:10.1371/journal.pone.0150273)
Supplement: S4 Fig — The bars indicate number of transcripts (left axis). The lines indicate N50 length (triangles) and average transcript length (rectangles) in bp (right axis). (PDF) [file pone.0150273.s004.pdf]

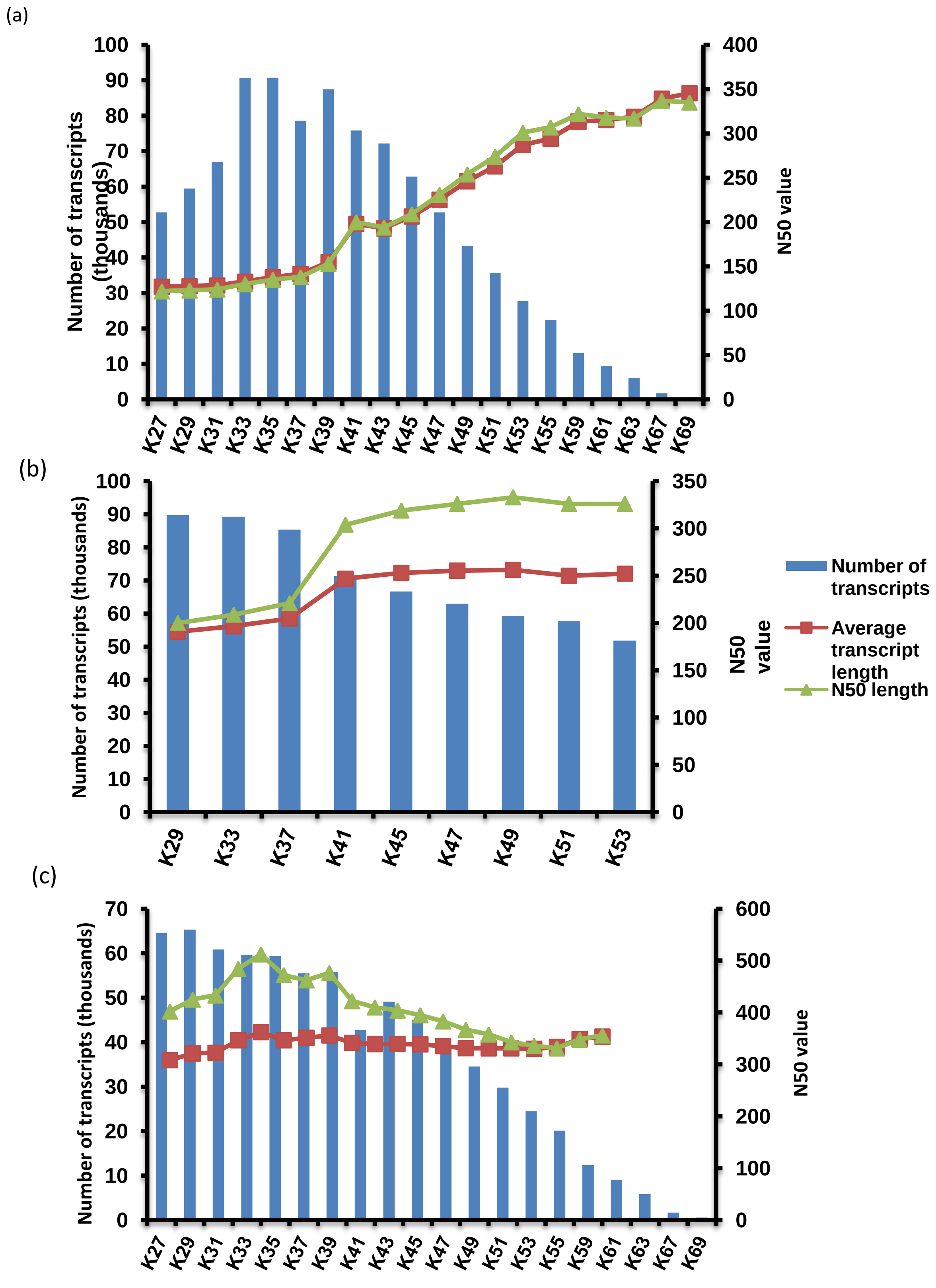

S4 Fig. Comparison of *de novo* assembly obtained through (a) Velvet, (b) ABySS and (c) Oases programs. The bars indicate number of transcripts (left axis). The lines indicate N50 length (triangles) and average transcript length (rectangles) in bp (right axis).
